# Genome wide transcriptional changes underlie gradual and recurrent adaptation to protein malnutrition in zebrafish

**DOI:** 10.1371/journal.pgen.1012071

**Published:** 2026-09-28

**Authors:** Siyao Wang, Laura Childers, Fernando Martinez III, Michel Bagnat, Jieun Park

**Affiliations:** 1 Department of Cell Biology, Duke University, Durham, North Carolina, United States of America; 2 Department of Molecular Genetics and Microbiology, Duke University, Durham, North Carolina, United States of America; 3 UNC Neuroscience Center, University of North Carolina at Chapel Hill, Chapel Hill, North Carolina, United States of America; 4 Department of Genetics, University of North Carolina at Chapel Hill, Chapel Hill, North Carolina, United States of America; University of Pennsylvania School of Medicine, UNITED STATES OF AMERICA

## Abstract

Lysosome-rich enterocytes (LREs) are a specialized population of intestinal cells that mediate the uptake and absorption of dietary proteins in fish and neonatal mammals. Loss of LRE function causes inhibited growth and poor survival due to protein malnutrition. Previously, we reported that in zebrafish loss of Plasmolipin (pllp), an endosomal membrane protein highly expressed in LREs, impairs LRE differentiation and dietary protein absorption, resulting in marked reduction in survival rates. Raising *pllp* homozygous mutants surviving to adulthood and in-crossing them for multiple generations resulted in a recovery of survival and growth, even when fed a low protein diet. To uncover mechanisms underlying this phenomenon, we compared the older adapted *pllp* allele with genetically related wild type (WT) fish and a newly generated *pllp* mutant allele. Using transcriptome profiling and quantitative protein absorption assays, we found that the adapted *pllp* mutants exhibit upregulation of LRE endocytic components associated with a capacity for protein absorption that exceeds that of WT. This hyperactivation of LRE endocytic and absorptive activity is accompanied by transcriptional regulation of immune genes that may contribute to the enhanced survival of *pllp* mutants in the face of increased exposure to environmental antigens. Genetic analyses indicate that these divergent phenotypes emerge gradually over generations and can recur in an independently generated *pllp* allele upon inbreeding and natural selection. Overall, our study illustrates that genome-wide transcriptional changes are associated with stable improvements in intestinal function and organismal survival in response to protein malnutrition.

## Author summary

In this study, we investigated how zebrafish adapt to protein malnutrition when the function of specialized intestinal protein-absorbing enterocytes, also found in newborn mammals, is impaired. We observed that fish carrying a mutation that severely disrupts intestinal protein absorption gradually recovered their ability to survive over multiple generations of inbreeding, even though the underlying mutation remained intact. By comparing gene activity in these adapted fish with that of newly generated mutants, we found that the adapted state is associated with changes in two systems: enterocytes show increased protein uptake and degradation that even surpass those of wild type fish, while the immune system was simultaneously recalibrated to dampen inflammation. We further show that this trajectory can be recapitulated in a second, independently generated mutant line that underwent a strikingly similar recovery trajectory over successive generations. Together, our findings reveal that animals can overcome a severe, heritable nutritional deficit through a gradual, genome-wide transcriptional response that fundamentally reshapes intestinal function across generations.

## Introduction

As an essential macronutrient, dietary protein is critical for animal development and for sustaining normal organismal physiology. Protein intake deficiencies, clinically manifested as stunting, wasting, and severe syndromes such as Kwashiorkor (nutritional edema), remain a major global health concern that contribute to pediatric morbidity and mortality [1,2]. Dietary protein absorption in zebrafish and neonatal mammals is highly dependent on lysosome-rich enterocytes (LREs), which are a group of specialized epithelial cells located in the ileum that are characterized by their giant lysosomal vacuoles and highly endocytic activity and capacity [3]. Previously described as vacuolated or neonatal enterocytes [4–6], LREs are widely conserved across various species of mammals including humans, rat, sheep, and pigs, as well as in teleost fishes [6–10]. While LREs are replaced by regular adult-type enterocytes upon weaning in mammals, they are retained through adulthood in fishes, which makes zebrafish an ideal model to study these cells that are only transient in the neonatal mammalian gut [3,11].

LREs absorb dietary protein via a multi-ligand complex machinery consisting of the endocytic adaptor protein Dab2, the transmembrane linker protein amnionless (Amn), and the scavenger receptor cubulin (Cubn) [3]. Loss of these components leads to poor survival, stunted growth in zebrafish and mice and also to intestinal edema reminiscent of Kwashiorkor disease in mice [3]. Apart from the endocytic machinery, the correct functioning of LREs also relies on endosomal protein plasmolipin (Pllp) [12]. Pllp is enriched at the apical region of epithelial Madin-Darby Canine Kidney (MDCK) cells during polarization in 3D cultures, where it is critical for maintaining apical sorting, recycling of SNAREs, and the activation of Notch signaling pathway [12]. In zebrafish, disruption of Pllp function in the *pllp*^*pd1116*^ mutant line resulted in impaired endocytic activity in LREs, leading to markedly reduced survival during larval stages [12].

Adaptation to nutrient scarcity occurs across two distinct timescales: the acute physiological response within a single individual and the transgenerational adaptation that shapes the fitness of subsequent generations. Severe acute malnutrition (SAM) and protein-energy malnutrition (PEM) in children and neonatal animal models are characterized by a shift to “protein-sparing” energy use, reduced growth, intestinal epithelial remodeling, endocrine changes, fatty liver, hypometabolism, and higher mortality during infections, which are often interpreted as adaptive attempts to survive at the cost of growth and immune competence [13–16]. At the core of adaptation are several well-studied nutrient-sensing and autophagy pathways including mammalian target of rapamycin (mTOR), AMP-activated protein kinase (AMPK), and General Control Non-depressible 2 (GCN2) [17–20]. Interestingly, in SAM or PEM animal models, refeeding alone often fails to return the animals to normal growth or cellular functions [13,14], suggesting that the acute episode leaves a durable physiological imprint on individuals. Previous studies demonstrated that such episodes remodel chromatin at autophagy and metabolic genes via DNA methylation and histone modifications, generating an epigenetic memory that is partially heritable [21,22]. Indeed, in murine models, offsprings produced by parents fed with low-protein diet (LPD) or with restricted access to dietary protein were shown to exhibit changes in promoter methylation and altered expressions of genes associated with metabolism, immune regulations, oxidative stress and neurodevelopment, with effects often strongest in F1 and decreasing to near normalization by F3 [23–26]. Most transgenerational studies on protein deficiency focus on manipulating wildtype animals using formulated low protein diets. In those studies, the transgenerational adaptation effects are largely attenuated by the F3 generation [23,26–29]. It was unclear how animals with inheritable genetic deficits that impair dietary protein intake could overcome a lethal survival disadvantage through lasting transgenerational adaptation that fundamentally changes the organ’s functional capacity.

Serendipitously, we found that raising surviving homozygous *pllp*^*1116*^ mutant zebrafish and subsequent inbreeding led to a loss of the survival defect originally reported in these mutants. Here, we investigate how these adapted *pllp* mutants could recover from a strong and largely lethal nutritional deficit via multigenerational adaptation and selection. We show that the F5 and subsequent generations of this zebrafish line show persistent changes in the transcriptional profiles of genes involved in protein absorption and immune responses that are in sharp contrast to that of newly generated *pllp* mutant alleles. Furthermore, using quantitative protein uptake and degradation assays, we show that the LREs in these adapted fish exhibit surprising hyperactivation of their protein endocytosis and degradation capabilities. Moreover, genetic experiments revealed that the adaptation to protein malnutrition is recurrent. Together, our results reveal that concurrent transcriptional regulations of multiple genes spanning LRE specific and as well as systemic processes underlie transgenerational adaptation to protein malnutrition in zebrafish.

## Results

### Zebrafish with impaired survival and reduced intestinal function due to the loss of *pllp* improve under multigenerational selection pressure

Previously, in Rodríguez-Fraticelli et al. (2015) we reported that zebrafish with a TALEN-generated nonsense mutation in *pllp* (expression shown in Fig 1A), named *pllp*^*pd1116*^*-/-,* showed severely impaired survival performance when raised with a limited food supply (AP100 commercial zebrafish diet; 80–90% reduction in survival rate), and exhibited dramatically reduced endocytosis of fluorescently labeled dextran in LREs. After creation of the original *pd1116*-/- stock, this genetic line was maintained via in-crossing of the surviving adult homozygous mutants. Following its generation, the in-cross offspring of F1 *pd1116*-/- was tested again for its survival performance under nutritional restriction using newly defined custom diets (Fig 1B) [30], to better model a protein-deficient challenge. Six days-post-fertilization (6 dpf) larvae were fed with either a calorie-restricted (10 mg food/tank/day) high-protein (HP) diet or calorie-restricted low-protein (LP) diet for 10 days until 16 dpf. Although the compromised survival phenotype originally reported in our previous study persisted when compared to the parent wild-type (WT) EK strain, the survival rate of those F2 *pd1116*-/- was noticeably higher than what we originally documented using undefined commercial diets (Fig 1C). Subsequently, the mutant strain was maintained as a homozygous mutant for 4 additional generations (Fig 1B). Then, to use them as reference in a new study, experimental in-crosses were generated and tested for survival using our defined diets and following the same calorie restriction scheme used previously. Surprisingly, the survival rate of F6 *pd1116*-/- offspring was comparable to EK under either diet condition (Fig 1D). Upon sequencing the *pllp* locus, we found no changes or loss to the original *pd1116*-/- mutation site compared to previously published data (S1A Fig).

**Fig 1:**
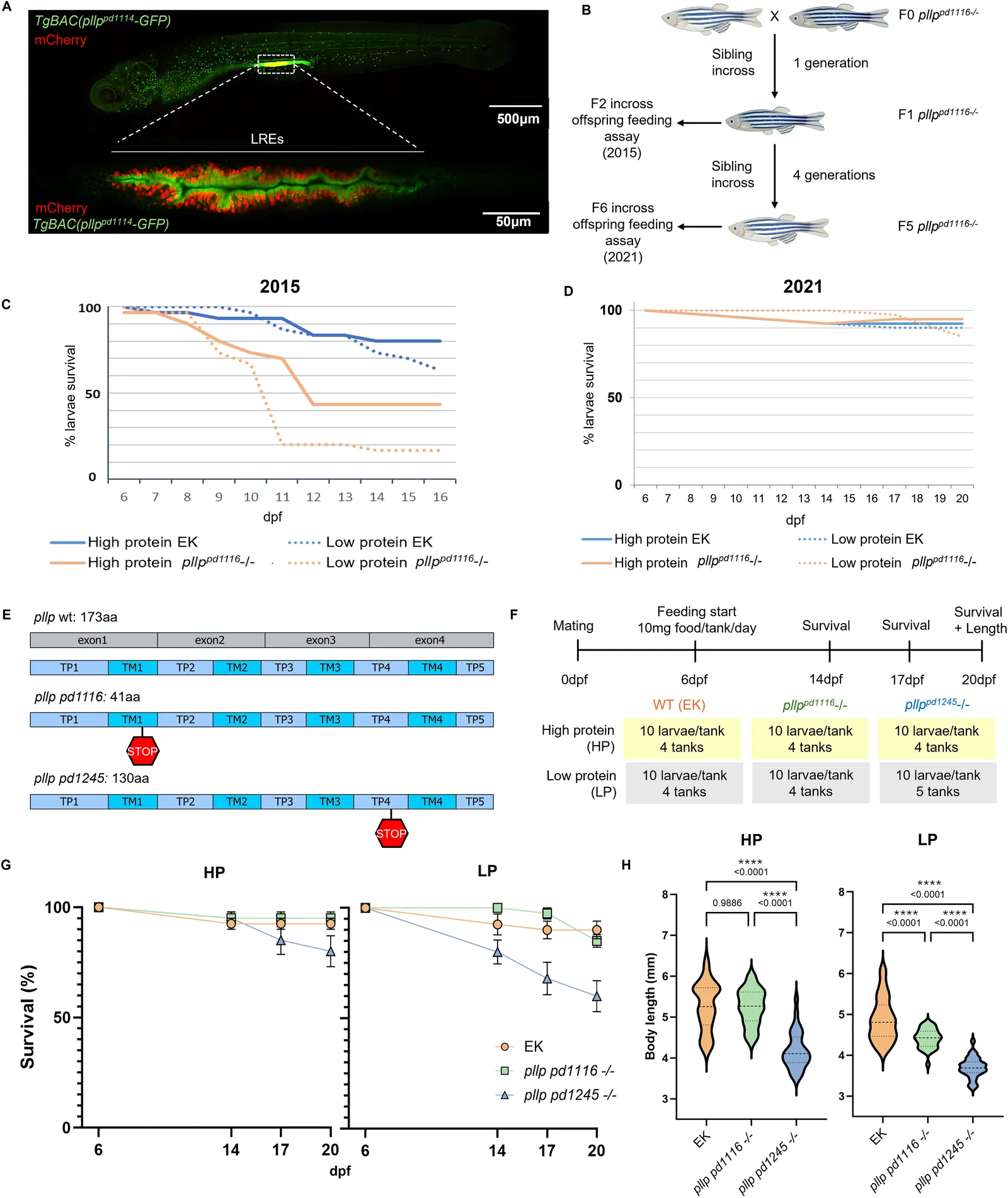
Increased survival of *pd1116*-/- under generational selection pressure. **(A)** Live image of a *TgBAC(pllp*^*pd1114*^*-GFP)* 6dpf larva gavaged with mCherry protein. The magnified area below is the LRE region. **(B)** Pedigree of the *pd1116* allele showing the generations used for feeding assays. Created in BioRender. W, S. (2026) https://BioRender.com/mgzlhdv **(C)** Survival curve of F2 *pd1116*-/- vs wild-type (EK) fed with calorie-restricted (10mg/tank/day) high-protein diet and calorie-restricted low-protein diet; experiment conducted in 2015; n = 4 tanks/condition/genotype, starting 10 larvae/tank. **(D)** Survival curve of F6 *pd1116*-/- vs EK conducted in 2021; n = 4 tanks/condition/genotype, starting 10 fish/tank. **(E)** Diagram of *pllp* alleles; *pd1116* allele was created by TALENs and has an early stop at amino aa41; *pd1245* was created by CRISPR-Cas9 using a single gRNA and has an early stop at aa130. TP = topological domain; TM = transmembrane domain. **(F)** Schematics of feeding experiment with calorie-restricted high-protein diet (HP) and calorie-restricted low-protein (LP) diet. **(G)** Survival curves of 3 genotypes (EK, F6 *pd1116*-/-, F4 *pd1245*-/-) over 20 days under HP or LP diet. n = 4 tanks/condition/genotype except for *pd1245*-/- LP (n = 5 tanks), starting 10 fish/tank. **(H)** Body lengths of surviving fish of 3 genotypes at 20dpf. n = 37 for EK HP; n = 38 for F2 *pd1116*-/- HP; n = 32 for F3 *pd1245*-/- HP; n = 36 for EK LP; n = 34 for *pd1116*-/- LP; n = 30 for *pd1245*-/- LP. One-way ANOVA.

To investigate the mechanisms underlying improved survival of *pd1116*-/-, we established a new loss of function (LOF) allele, *pd1245,* to mimic *pd1116*-/- in its pre-adapted state. Given the lack of suitable CRISPR target sites in exon1, which harbors the original *pd1116* TALEN-generated mutation, *pd1245* was created using a single CRISPR gRNA targeting exon 2 of *pllp*, resulting in a 10 base pair (bp) deletion which produces a nonsense mutation downstream at amino acid 130 and a truncation of transmembrane domain 4 (Figs 1E and S1B). To characterize the new *pd1245* allele, we first performed RT-PCR to examine transcript expression levels and found that both *pd1245* and *pd1116* alleles exhibit significant degradation of the *pllp* transcript as compared to WT EK, suggesting that non-sense mediated decay (NMD) occurs as a result of the presence of premature stop codons (S1C, S1C’ Fig).

Next, we conducted a 20-day feeding experiment using a calorie-restricted HP or LP diet design. At 6 dpf, larvae from in-cross matings of EK, *pd1116*-/- or *pd1245*-/- were sorted into 4 or 5 tanks per genotype per diet condition at a density of 10 larvae per tank. Each tank received 10 mg of HP or LP diet per day until 20 dpf. The number of surviving larvae per tank were counted at 14, 17, and 20 dpf to record survival performance. Standard length [31] of any remaining larvae at 20 dpf was measured as proxy of growth (Fig 1F). We found that the average survival rate of *pd1245*-/- larvae declined to 80% under HP and 60% under LP, as compared to 92.5% (HP) and 90% (LP) for EK or 95% (HP) and 85% (LP) for *pd1116*-/- (Fig 1G). Furthermore, body growth of *pd1245*-/- larvae was significantly stunted (4.18mm HP, 3.70mm LP) as compared to both EK (5.26mm HP, 4.88mm LP) or *pd1116*-/- (5.25mm HP, 4.41mm LP) under either feeding condition (Fig 1H). Together, these data show that *pd1245*-/- partially reproduced the impaired survival phenotype and could be used as an approximation of the *pd1116*-/- in its pre-adapted state comparative studies.

### Transcriptomic profiling reveals genotype-dependent and diet-dependent gene expression programs in *pllp* alleles

To identify transcriptomic changes associated with *pllp* loss and adaptation, we performed bulk RNA sequencing on 20 dpf whole zebrafish larvae from three genotypes (EK, adapted *pd1116*-/-, and newly generated *pd1245*-/-) and feeding conditions described in Fig 1F. For each genotype/diet condition, three tanks were randomly selected as independent replicates; larvae from each tank were pooled prior to RNA extraction, yielding one pooled sample per replicate. Hierarchical clustering of all expressed genes revealed distinct transcriptional profiles across genotype and diet conditions (Fig 2A). To investigate whether alternative splicing or restored transcript variants contributed to *pd1116-/-*’s transcriptomic recovery, we analyzed exon-level usage from our RNA-seq dataset. Integrative Genomics Viewer (IGV) visualization of splice junction coverage combined with Differential Exon Usage Sequence (DEXseq) analysis of relative exon usage at the *pllp* locus revealed the absence of novel splice isoforms or exon skipping, ruling out alternative splicing as a driver of *pd1116*-/-’s improved survival (S1D-S1F Fig). Principal component analysis (PCA) of global gene expression patterns showed that PC1 separated samples primarily by genotype, with *pd1116*-/- clustering closer to EK than to *pd1245*-/-, consistent with transcriptional normalization occurring in the adapted *pd1116*-/- strain (Fig 2B). Notably, PC1 also captured diet-dependent separation within mutant genotypes as both adapted *pd1116*-/- and newly-generated *pd1245*-/- showed clear separation between HP and LP conditions, whereas EK HP and LP samples clustered together. This indicates that while diet has minimal impact on WT transcription, it profoundly influences gene expression in both *pllp* mutant backgrounds. PC2 further resolved genotype-by-diet interactions, separating *pd1116*-/- and *pd1245*-/- into distinct transcriptional states under each dietary condition (Fig 2B).

**Fig 2:**
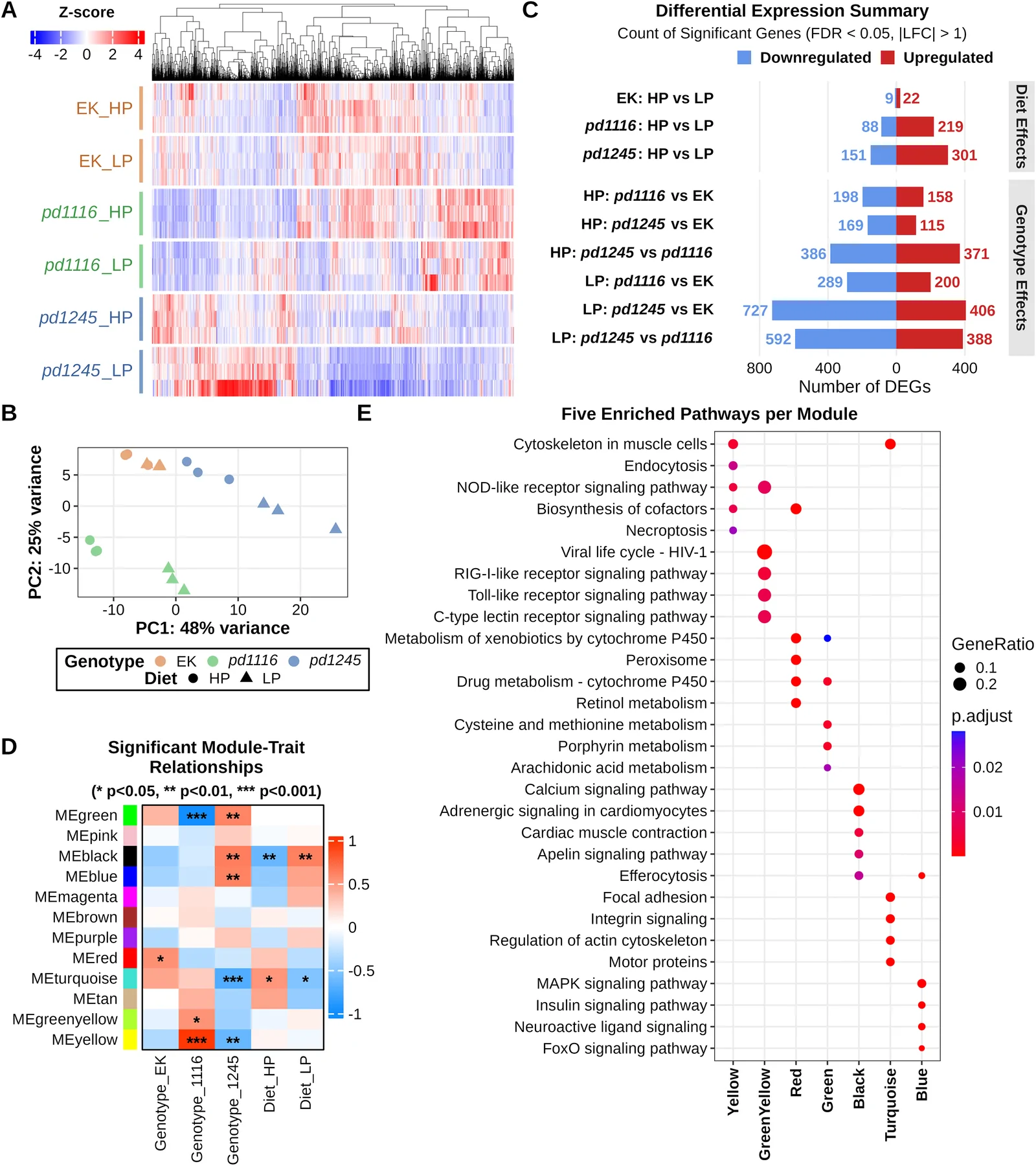
Transcriptomic analyses of WT and *pllp* alleles raised with HP and LP diets. Weighted gene co-expression network analysis (WGCNA) reveals genotype-specific transcriptional signatures. **(A)** Heatmap showing expression of the 2,279 genes called differentially expressed in at least one of the nine pairwise comparisons in **(C)** (FDR < 0.05, |log2 fold-change| > 1). Rows are 18 samples, grouped by genotype x diet; columns are genes. Color scale indicates relative expression level (per-gene z-score). **(B)** Principal component analysis (PCA) of global gene expression. PC1 separates genotypes, with *pd1116-/-* positioned closer to EK than *pd1245-/-*, reflecting transcriptional normalization. EK samples cluster together regardless of diet, while *pd1116-/-* and *pd1245-/-* show diet-dependent separation along PC1 and genotype-by-diet interaction along PC2. **(C)** Summary of differentially expressed genes (DEGs; adjusted p < 0.05, |log2FC| > 1) across all pairwise comparisons. Bar lengths indicate DEG counts; colors distinguish upregulated (red) and downregulated (blue) genes. EK shows minimal diet-responsive DEGs [30], while *pd1245-/-* under LP shows the largest transcriptional disruption (1,133 DEGs vs. EK). See also S1 Table. **(D)** Module-trait relationship heatmap showing Pearson correlations between module *eigengenes* and experimental traits (genotype: EK, *pd1116-/-*, *pd1245-/-*; diet: HP, LP). Color scale indicates correlation strength (red, positive; blue, negative). Four modules of interest are Yellow (positively correlated with *pd1116*, p = 3.77 × 10^−12^), Green (negatively correlated with *pd1116-/-*, p = 4.99 × 10^−14^), Black (positively correlated with *pd1245-/-* and LP diet), and Turquoise (negatively correlated with *pd1245-/-*, positively correlated with HP diet). **(E)** Five significantly enriched KEGG pathways per module (selected by gene overlap). Dot size indicates GeneRatio (fraction of module genes in the pathway); color indicates BH-adjusted p-value; all modules included show significance in module-trait relationships.

To identify transcriptional differences driven by genotype and diet, we performed differential expression analysis using DESeq2 testing for both main effects and genotype-by-diet interactions (*genotype + diet + genotype:diet*). We conducted nine pairwise comparisons: diet effect within each genotype (HP vs. LP for EK, *pd1116-/-*, and *pd1245-/-*), and genotype effects within each diet condition (EK vs. *pd1116-/-*, EK vs. *pd1245-/-*, and *pd1116-/-* vs. *pd1245-/-*, separately for HP and LP). DEGs were defined by False Discovery Rate (FDR) < 0.05 and |log_2_ fold change| > 1. We identified markedly different numbers of differentially expressed genes (DEGs) across comparisons (Fig 2C and S1 Table). Within each genotype, EK showed only 31 DEGs between HP and LP diets (22 upregulated, 9 downregulated), confirming that diet minimally perturbs wild-type transcription. In contrast, *pd1116*-/- showed 307 diet-responsive DEGs (219 up, 88 down) and *pd1245*-/- showed 452 (301 up, 151 down), suggesting that *pllp* loss sensitizes larvae to dietary perturbation. When comparing mutants to EK, *pd1116*-/- showed 356 DEGs under HP (158 up, 198 down) and 489 under LP (200 up, 289 down), while *pd1245*-/- showed 284 DEGs under HP (115 up, 169 down) but 1,133 under LP (406 up, 727 down), which represents a dramatic expansion of transcriptional disruption under nutritional stress. The direct comparison of *pd1245*-/- versus *pd1116*-/- yielded 757 DEGs under HP and 980 under LP, underscoring the substantial transcriptional divergence between the adapted mutant line and the newly generated allele.

To identify genotype-specific transcriptional signatures, we examined the overlap of diet-responsive and genotype-responsive DEGs across conditions. Comparison of diet effects (HP vs. LP) across genotypes revealed that the majority of EK diet-responsive DEGs were conserved in the mutant backgrounds: out of the 31 EK diet-responsive DEGs in HP vs LP, 19 (61%) overlapped with *pd1116*-/- diet-responsive genes, and 11(35%) overlapped with *pd1245*-/- (S2A Fig and S2 Table). This indicates that *pllp* mutants retain the core wild-type transcriptional response to dietary protein restriction while acquiring hundreds of additional genotype-specific diet-responsive genes expression changes (197 unique to *pd1116*-/-; 350 unique to *pd1245*-/-), reflecting a dramatically expanded sensitivity to nutritional perturbation. The larger overlap between EK and *pd1116*-/- than between EK and *pd1245*-/- is consistent with *pd1116*-/- transcriptional normalization toward the WT state. Comparison of genotype effects within each diet condition revealed distinct and overlapping gene sets between *pd1116*-/- and *pd1245*-/- relative to EK (S2B, S2C Fig). Under HP conditions, 237 genes were uniquely dysregulated in *pd1245*-/- versus EK but not in *pd1116*-/- versus EK, representing a potentially pathological profile specific to the non-adapted mutant. Under LP conditions, this *pd1245*-unique gene set expanded substantially, consistent with the exacerbated survival defect of *pd1245*-/- under nutritional stress. Conversely, genes uniquely dysregulated in *pd1116*-/- but not *pd1245*-/- represent candidate transcriptional signatures underlying the divergent survival phenotypes.

### Weighted gene co-expression network analysis reveals transcriptional programs associated with the adapted *pd1116*-/- state

To capture coordinated transcriptional programs, we performed weighted gene co-expression network analysis (WGCNA) [32], which groups genes into modules of correlated expression and tests whether each module’s eigengene correlates with experimental traits. We constructed a signed hybrid coexpression network using all 18 samples (three genotypes × two diets × three replicates), allowing us to ask simultaneously which modules associate with genotype, with diet, or with both. Module eigengenes (MEs), which represent the first principal component of each module’s expression profile, were then correlated with genotype and diet traits using Pearson correlation to identify modules most relevant to the experimental conditions. Because co-expression modules capture correlation rather than direct regulation, these associations are interpreted as candidate transcriptional programs linked to the adapted state rather than as established causal pathways. WGCNA identified 12 co-expression modules (excluding the grey module of unassigned genes). Module-trait correlations were then tested for genotype (EK, *pd1116-/-*, *pd1245-/-*) and diet (HP, LP) to prioritize modules most likely to reflect biologically meaningful transcriptional responses to these variables. Of 12 modules, seven of them (Green, Black, Blue, Red, Turquoise, Greenyellow, Yellow) showed statistically significant associations with genotype and/or diet (Figs 2D, S2D).

The Yellow and Green modules exhibited opposing transcriptional profiles in relation to genotype; while the Yellow module was significantly positively correlated with the *pd1116*-/- genotype, the Green module showed a strong negative correlation (Figs 2D, S2D). Notably, neither module showed significant association with diet (Yellow: p = 0.77; Green: p = 0.99), indicating that the transcriptional shifts captured by these modules are constitutively maintained in *pd1116-/-* regardless of nutritional status. This diet-independence distinguishes them from the broader, environmentally-sensitive transcriptional changes and suggests that these specific compensatory (Yellow) and suppressive (Green) programs represent constitutively shifted transcriptional states in the adapted *pd1116*-/- line, which may have become stabilized over generations of homozygous maintenance. Beyond genotype-specific effects, several modules exhibited sensitivity to both genetic background and dietary conditions. The Black and Turquoise modules showed a conserved response to diet across all genotypes; the Black module was consistently induced under LP conditions, whereas the Turquoise module showed the opposite, downward trend under LP status. Interestingly, the Blue module revealed a genotype-specific dietary sensitivity, as its expression remained largely stable across most conditions but spiked specifically in *pd1245-/-* LP condition. Finally, while the Red and Greenyellow modules also reached statistical significance, they exhibited less consistent direction trends across the experimental groups (Figs 2D, S2D).

KEGG pathway enrichment of module genes revealed distinct functional signatures for each module (Fig 2E). The Yellow module (upregulated in *pd1116*-/-) was enriched for cytoskeleton in muscle cells, endocytosis, NOD-like receptor signaling, and biosynthesis of cofactors. Together, these pathways suggest that *pd1116*-/- enhanced survival phenotype may be associated with cytoskeletal reinforcement, enhanced endocytosis, activation of innate immune responses, and upregulation of metabolic programs.

The Green module (downregulated in *pd1116*-/-) was enriched for drug metabolism via cytochrome P450, porphyrin metabolism, cysteine and methionine metabolism, and arachidonic acid metabolism (Fig 2E). This suggests that *pd1116*-/- may have selectively suppressed certain detoxification and lipid-sensing programs.

The Turquoise module’s eigengene expression was reduced under LP to HP diet across all three genotypes (S2D Fig). However, the magnitude of this downregulation was markedly greater in the *pllp* mutants (*pd1116* and *pd1245*). Notably, the baseline expression of this module in the *pd1245* mutants under HP conditions was suppressed to levels similar to the LP-treated *pd1116* group (S2D Fig). Functional enrichment of the Turquoise module highlighted pathways related to the muscle cell cytoskeleton, focal adhesion, integrin signaling, regulation of the actin cytoskeleton, and motor proteins (Fig 2E). This suggests that the LP condition impairs muscle structural integrity and growth, and that this physical deficit is much more severe in the *pllp* mutants than in the EK controls.

Conversely, both the Blue and Black modules exhibited increased eigengene expression under the LP diet relative to HP, a pattern that was profoundly amplified in the *pllp* mutants (S2D Fig). While the Blue module showed a dramatic upregulation in the *pd1245* LP group, the Black module demonstrated an even greater overall diet-induced shift, with the differential expression between HP and LP states vastly exacerbated in both mutant lines compared to the EK controls (S2D Fig). The Blue module was enriched for MAPK signaling, insulin signaling, and neuroactive ligand-receptor interactions, suggesting a metabolic stress and nutrient-sensing response under LP condition in *pd1245-/-* (Fig 2E) [33–36]. Concurrently, the Black module was enriched for the calcium signaling pathway, adrenergic signaling in cardiomyocytes, cardiac muscle contraction, and apelin signaling (Fig 2E). Together, these profiles suggest that *pllp* mutants under LP condition undergo heightened physiological stress in the absence of Pllp function.

### *pllp* loss of function disrupts lipid metabolism, extracellular matrix integrity, and cell cycle progression

To identify the biological pathways disrupted by the loss of *pllp* only in a newly generated mutant, we performed KEGG pathway analysis on DEGs unique to *pd1245*-/- (non-overlapping with *pd1116*-/-) relative to EK, under both HP and LP conditions (S3A Fig). By excluding DEGs shared between both mutants which represent *pllp*-associated changes that persist regardless of adaptation, this strategy enriches for pathways that are disrupted in the newly generated *pd1245*-/- but have been restored to near-normal expression levels in adapted *pd1116*-/-.

Genes uniquely downregulated in *pd1245*-/- under LP conditions showed strong enrichment for vitamin digestion and absorption, steroid biosynthesis, phagosome, cell cycle, and ECM-receptor interaction pathways. Under HP conditions, uniquely downregulated genes in *pd1245*-/- were enriched for biosynthesis of unsaturated fatty acids, fatty acid metabolism, and cytoskeleton in muscle cells (S3A, S3B Fig). Together, these results suggests that *pllp* loss leads to a collapse of lipid and cholesterol metabolism, disruption of extracellular matrix organization, and inhibition of cell proliferation, with the severity of these defects amplified under LP conditions (S3A Fig).

To examine gene expression patterns of the lipid related DEGs (unique to *pd1245*-/- vs EK) across all experimental groups, we visualized the expression of some of the DEGs in these terms via heatmap (S3B Fig). Genes involved in lipid and cholesterol metabolism as well as vitamin digestion and absorption, including the endocytic receptor genes cubulin (*cubn*) and amnionless (*amn*), which mediate nutrient uptake in LREs [3], were markedly downregulated in *pd1245*-/- particularly under LP conditions. In *pd1116*-/-, however, these genes were largely normalized to EK levels under HP conditions, with moderate downregulation under LP that was substantially less severe than in *pd1245*-/-. These results suggest that the pathways most disrupted by acute *pllp* loss has partially normalized in the adapted *pd1116*-/- line, consistent with broader transcriptional reorganization associated with the adapted state.

### *pd1116-/-* exhibits enhanced expression levels of LRE endocytic machinery genes and protein uptake kinetics

To investigate potential cell type changes linked to the adaptation of *pd1116*-/-, we first examined the expression of well-known genetic markers of various intestinal cell groups [37]. Interestingly, at 20 dpf following the feeding assay, *pd1116*-/- did not show consistent upregulations of markers for intestinal cell types important for nutrient sensing or absorptions including absorptive enterocytes, LREs and enteroendocrine cells compared to EK or *pd1245*-/- (S4A Fig). We next quantified the number of active LREs by gavaging dextran 568 and segmenting the vacuoles in Ilastik and found that *pd1116*-/- did not develop more active LREs compared to EK or *pd1245*-/- (S4B-S4D Fig. Interestingly, *pllp* LOF in *pd1245*-/- did not seem to disrupt LRE differentiation as evidenced by the comparable vacuole number to EK (S4D Fig).

As mentioned above, our WGCNA and KEGG pathway analyses suggest a strong positive correlation of genes involved in endocytosis with *pd1116*-/-, including *amn* and *cubn*, which encode components of the scavenger receptor complex Cubulin-Amionless. These upregulated genes suggested a role for increased LRE function in *pd1116*-/-’s adaptation from a malnourished state. Indeed, in *pd1116*-/- many genes associated with endocytic machinery, proteases and lysosome hydrolases have comparable expressions to EK but significantly higher expression levels than *pd1245*-/-, especially under LP condition (Fig 3A, 3B), including the voltage-sensing phosphatase gene *tpte* which is also required for protein uptake in LREs [3,38,39]. To test whether this observed pattern at 20 dpf is established at an earlier developmental stage before the onset of external feeding, we conducted HCR in situ hybridization against *dab2*, *cubn*, and *amn* in 6 dpf larvae. At 6 dpf, *pd1116*-/- showed expression levels of all three LRE endocytic machinery genes that were significantly higher than those of both EK and *pd1245*-/- (Fig 3C-3E”). To investigate if such upregulation of LRE endocytosis translates to increased protein absorption, we performed a protein uptake and degradation kinetics assay in 6 dpf larvae using fast degrading mTurquoise as protein cargo (Fig 4A). We found that *pd1116*-/- was able to both take up significantly more mTurquoise protein cargo within 30 min post-gavage and degrade them much faster than EK or *pd1245-/-* over a 60 min period (Fig 4B-4E). Together, these data show that transcriptional upregulations of key LRE endocytic machinery genes, rather than changes in LRE numbers, is closely associated with the higher protein absorption capacity observed in the adapted *pd1116*-/- line, although the precise genetic mechanisms underlying this improvement remain to be defined.

**Fig 3:**
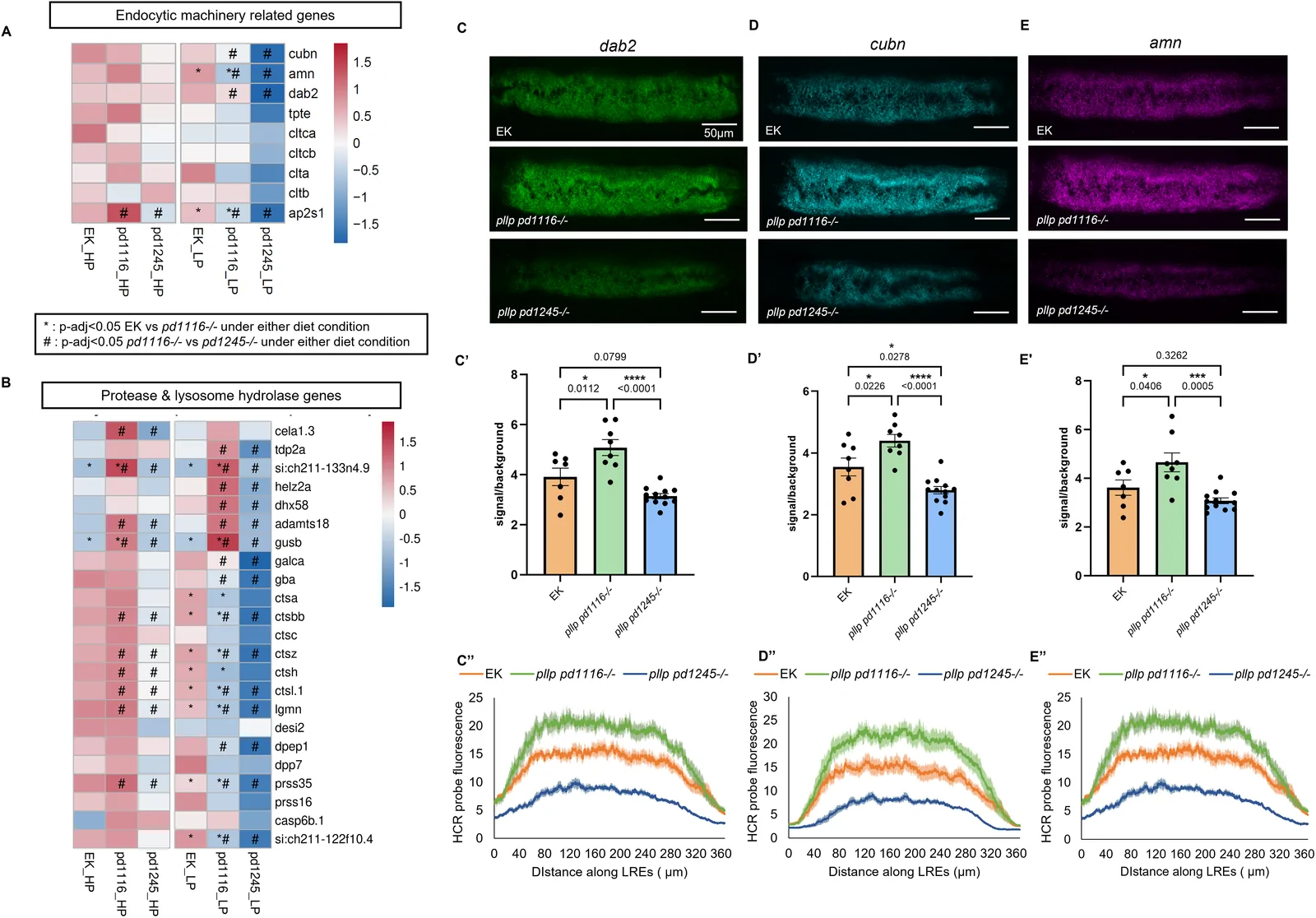
Upregulated transcription of endocytic machinery, protease, and lysosome hydrolase genes in *pd1116-/-.* **(A)** Gene expression heatmap of endocytic machinery related genes selected from bulk RNA-seq data. **(B)** Gene expression heatmap of protease and lysosome hydrolase related genes selected from bulk RNA-seq data. Values are per-gene z-scores of variance-stabilized counts averaged within genotype x diet group. Markers denote DESeq2 significance within the indicated diet: * = FDR < 0.05 for EK vs *pd1116* (marking both the EK and *pd1116* cells of that pair); # = FDR < 0.05 for *pd1116* vs *pd1245* (marking both the *pd1116* and *pd1245* cells). **(C-E”)** Representative images, signal profile and expression level quantification of HCR in situ hybridization for *dab2* (C-C”), *cubn* (D-D”) and *amn* (E-E”) in 6dpf EK, F7 *pd1116-/-,* F5 *pd1245-/-,*; n = 7 for EK, n = 8 for *pd1116-/-,* n = 12 for *pd1245-/-*; one-way ANOVA.

**Fig 4:**
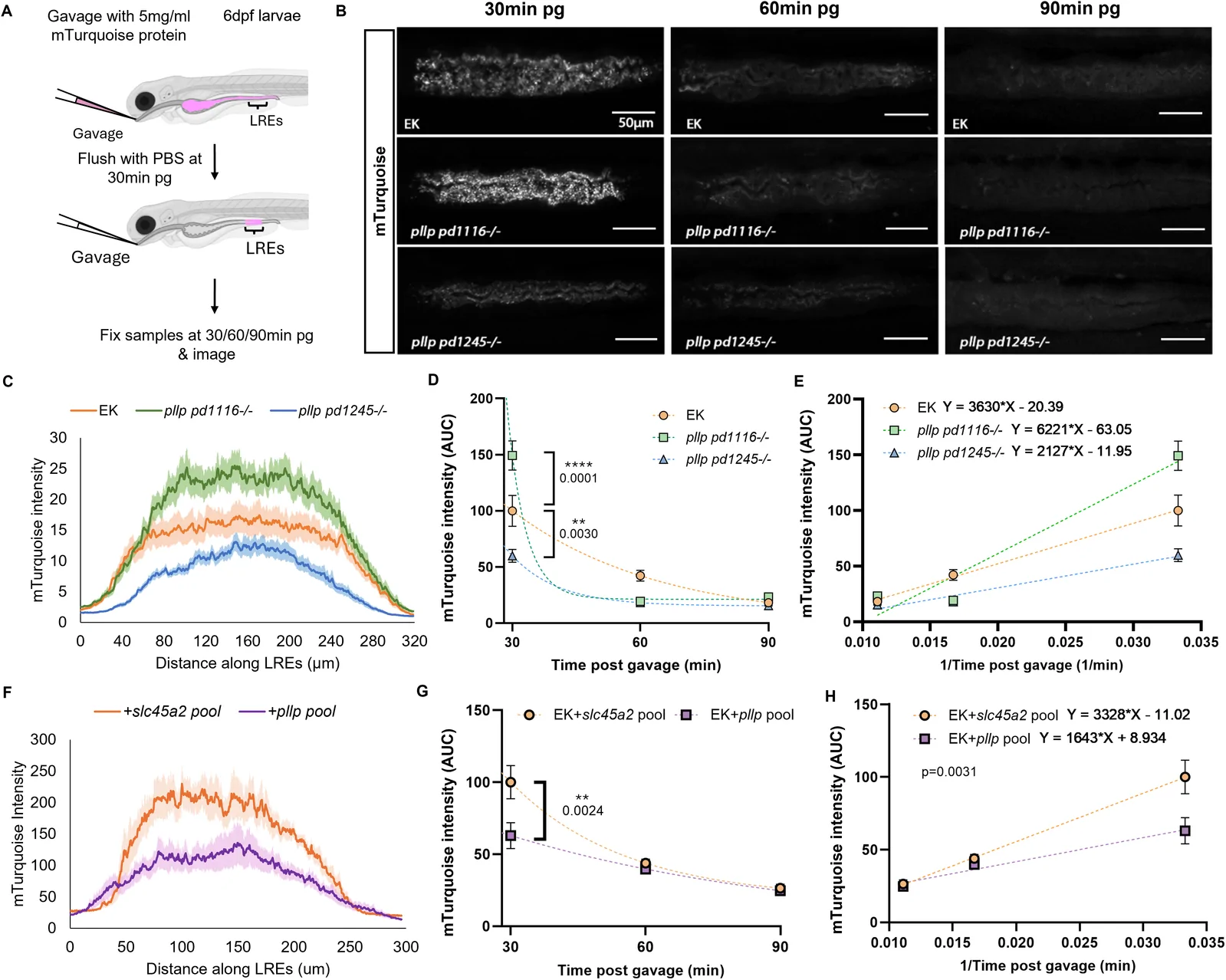
*pd1116-/-* shows enhanced protein uptake and degradation kinetics. **(A)** mTuquoise protein gavage assay experimental design; experiment were performed in 6 dpf EK, F7 *pd1116-/-,* and F5 *pd1245-/-* larvae. Created in BioRender. W, S. (2026) https://BioRender.com/mgzlhdv **(B)** Representative images of the LRE region at 30/60/90min post gavage (pg). **(C)** mTurquoise fluorescent signal profile at 30min pg. **(D, E)** Quantifications of mTurquoise fluorescent level curves at 30/60/90min pg (D) and linearly transformed curves showing degradation rates of each genotype **(E)**; 30min pg: n = 15 for EK, n = 16 for *pd1116-/-*, n = 17 for *pd1245-/-*; 60min pg: n = 15 for EK, n = 15 for *pd1116*-/-, n = 13 for *pd1245-/-*; 90min pg: n = 13 for EK, n = 14 for *pd1116*-/-, n = 14 for *pd1245-/-;* data normalized to EK 30min pg; 2-way ANOVA & Tuky’s test **(D)** ANCOVA: p < 0.001 (*pd1116-/-* vs *pd1245-/-*), p < 0.001 (*pd1116-/-* vs EK), p = 0.2728 (*pd1245-/-* vs EK) **(E)**. **(F)** mTurquoise fluorescent signal profile showing decreased uptake in *pllp* gRNA pool CRISPR respect to *slc45a2* control gRNA pool EK at 30min pg; n = 8 for EK + *slc45a2* pool gRNA, n = 10 EK+*pllp* pool gRNA. **(G, H)** Quantifications of mTurquoise fluorescent level curves at 30/60/90min pg (G) and linearly transformed curves showing degradation rates of each genotype **(H)**; 30min pg: n = 8 for EK + *slc45a2* pool gRNA, n = 10 for EK+*pllp* pool gRNA; 60min pg: n = 8 for EK + *slc45a2* pool gRNA, n = 8 for EK+*pllp* pool gRNA; 90min pg: n = 8 for EK + *slc45a2* pool gRNA, n = 8 for EK+*pllp* pool gRNA; data normalized to EK + *slc45a2* pool gRNA 30min pg; 2-way ANOVA & Tuky’s test **(G)**; ANCOVA **(H)**.

Our quantitative protein absorption assay confirmed that loss of Pllp function in *pd1245*-/- leads to reduced protein absorption by LREs. We then created a new *pllp* LOF allele, *pd130*9*,* using a single CRISPR gRNA targeting TM2. *pd1309* carries an in-frame deletion of 21 bp in exon 2, resulting in a 35% shorter TM2, predicted to disrupt membrane insertion without causing *pllp* transcript decay (S5A-S5C’ Fig). Upon assaying protein absorption, we found that the *pd1309-/-* larvae showed a significant reduction in mTurquoise protein level at 30 min post-gavage compared to EK, similar to what we observed in *pd1245*-/- and the *pllp* CRISPR-Cas9 pool KD group (S5D, S5E Fig).

To provide an allele‑independent and acute experimental validation of Pllp function, we generated a CRISPR-Cas9 gRNA pool consisting of 4 gRNAs targeting exons 1–3 of *pllp,* and a control gRNA pool targeting of the pigment gene solute carrier family 45 member 2 (*slc45a2)* [40]. We validated the knock down (KD) efficiency of the *pllp* CRISPR pool by injecting into heterozygous *TgBAC(pllp*^*pd1114*^*-GFP)*, a bacterial artificial chromosome (BAC) reporter line that expresses Pllp-GFP under the control of the *pllp* regulatory sequences (Fig 1A). This resulted in a complete loss of GFP+ larvae at 6dpf as opposed to the 43.96% population of GFP+ larvae in non-injected control group, suggesting depletion of Pllp at both the transcript and protein levels (S5F Fig). We also validated the *slc45a2* gRNA pool as a control by comparing the body length and mTurquoise uptake capability at 30min post-gavage to non-injected EK. *slc45a2* injected larvae showed an almost complete loss of pigmentation, as well as body length and mTurquoise uptake levels comparable to non-injected EK, showing that *slc45a2* KD is a suitable control for LRE experiments (S5G–S5J Fig). Injection of Cas9 gRNA and the *pllp* gRNA pool into EK led to a significant reduction in both protein uptake and degradation kinetics compared to *slc45a2* KD controls (Fig 4F-4H). These data demonstrate that impaired LRE protein absorption is a general and immediate consequence of Pllp loss, independent of the precise genomic lesion or genetic background.

These data demonstrate that either acute loss of *pllp* function (CRISPR KD), non-sense mutant alleles (*pd1245*), or mutated alleles not subject to NMD (*pd1309*) can reproduce the protein absorption deficits of the original *pllp* mutation *pd1116* before its adaption.

Taken together, our data firmly establishes a role for *pllp* in LRE function and suggests that *pd1116*-/- developed improved LRE protein absorption capacity. This LRE hyperactivity may allow it to maximize the utilization of dietary protein under restrictive conditions, crucially contributing to its generational adaptation to protein malnutrition.

### *pd1116* adaptation involves fine-tuned expression of innate and adaptive immune response genes

Further examination of our RNA sequencing data GO enrichment on *pd1116 vs EK* DEGs under either diet condition revealed transcriptional changes in genes associated with response to bacterium as well as immune response (Fig 5A, 5B). Under HP condition, immune response DEGs had the highest relative expressions in *pd1116-/-* compared to EK, while they were downregulated in *pd1245*-/- (Fig 5A). The lowered relative expressions of DEGs in the same category in *pd1245*-/- than EK remained true under LP condition. Meanwhile, DEGs associated with response to bacterium were lower in *pd1116*-/- than EK under both diet conditions. These observations suggest that *pd1116*-/- may have become less reactive to bacterial stimuli, while remaining immunologically active.

**Fig 5:**
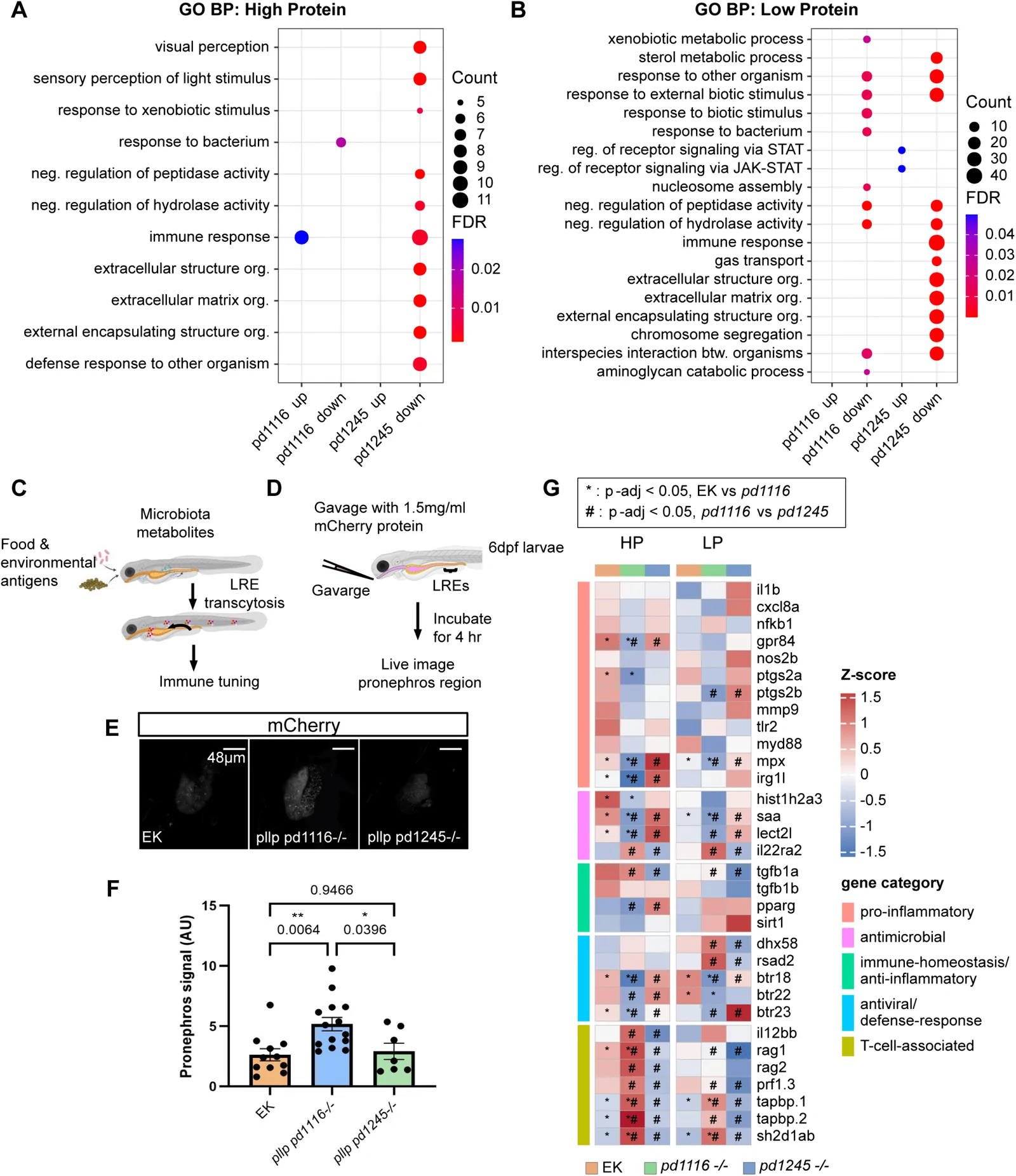
Innate and adaptative immune response adaptations in *pd1116-/-.* **(A, B)** GO Biological Processes enrichment of genes differentially expressed versus EK, shown separately for (A) high-protein and (B) low-protein. Columns are the four gene sets per diet (*pd1116* and *pd1245*, up- and down-regulated versus EK); rows are enriched GO BP terms. Columns without dots had no significant term. Dot size, number of genes; color, BH FDR. **(C)** Cartoon showing the proposed model of how food and environmental antigens as well as microbiota metabolites could trigger immune tuning via LRE transcytosis. Created in BioRender. W, S. (2026) https://BioRender.com/mgzlhdv **(D)** mCherry transepithelial transcytosis experiment setup. Created in BioRender. W, S. (2026) https://BioRender.com/mgzlhdv **(E)** Representative images of the pronephros region mCherry signal at 4h pg for EK, F7 *pd1116-/-* and F5 *pd1245-/-* and **(F)** quantifications of the signals; n = 11 for EK, n = 14 for *pd1116-/-,* n = 7 for *pd1245-/-;* one-way ANOVA. **(G)** Gene expression heatmaps of 32 curated immune genes, grouped into five functional categories (colour bar, left; key, right). Values are the per-gene z-score of variance-stabilised counts across all 18 samples, averaged within each genotype × diet group. Columns are grouped by diet (HP, LP) and coloured by genotype (key below the panel). Markers denote DESeq2 significance within the indicated diet: * = FDR < 0.05 for EK vs *pd1116* (marking both the EK and *pd1116* cells of that pair); # = FDR < 0.05 for *pd1116* vs *pd1245* (marking both the *pd1116* and *pd1245* cells).

Previously we documented LRE-mediated transcytosis of orally gavaged mCherry protein and transport via circulation to the pronephros, an kidney-like organ in zebrafish, via circulation [3,41]. This phenomenon was particularly intriguing as it was reminiscent of the well-documented luminal antigen transcytosis process by M cells, enterocytes and goblet cells in mammals that helps balance immune activation with tolerance [42–44]. Based on the heightened LRE protein uptake level and the more active immune responses in *pd1116*-/-, we hypothesize that *pd1116*-/- might have fine-tuned its immune system to better accommodate increased level of foreign antigens transcytosed by LREs (Fig 5C). To test whether *pd1116*-/- exhibits increased transcytosis, we gavaged 6 dpf larvae with mCherry protein cargo and quantified the signal intensity in the pronephros(Fig 5D). At 4 h post-gavage, *pd1116*-/- exhibited significantly more mCherry signal in the pronephros than both EK and *pd1245*-/- (Fig 5E, 5F).

Close examination of DEGs within GO term pathways for immune responses and response to bacterium, as well as well-known immune genes revealed an intricate balance of immune system changes (Fig 5G). in *pd1116*-/-, we observed a general suppression of pro-inflammatory genes, downregulation of antimicrobial responses, and careful balancing of anti-inflammatory and anti-viral defense response reactions in *pd1116*-/- compared to EK or *pd1245*-/-, especially under HP condition. Interestingly, these gene regulation patterns also suggest a possible premature onset of adaptive immune maturation by 20 dpf, earlier than the literature-documented 28–42 dpf [45]. This is supported by the significant upregulation of several T-cell function associated genes including *interleukin 12B, b* (*il12bb)*, *recombination activating gene 1* (*rag1)*, r*ecombination activating gene 2 (rag2), perforin-1.3 (prf1.3), TAP binding protein 1 (tapbp.1), TAP binding protein 2 (tapbp.2),* and *SH2 domain containing 1A duplicate a (sh2d1ab)* in *pd1116*-/- compared to EK or *pd1245*-/- under HP condition (Fig 5G). Combined with our previous results of LRE hyperactivity, we reason that this immune response may play a role in preventing inappropriate inflammatory reactions to internalized protein macromolecules or bacterial metabolites. Such regulation may be particularly critical under malnourished conditions, as inflammations are generally considered energetically costly [46–48].

Together, these data suggest that exacerbated immune activity and inflammation may play a role in the demise of *pd1245*-/- fish, particularly under LP conditions. Conversely, a better tuned immune response in *pd1116*-/- may allow them to meet their nutritional needs while avoiding excessive inflammation caused by increased trafficking of microbial and dietary antigens. This may enable them to tolerate the significant increase in transepithelial transport across the intestine, likely as a byproduct of increased endocytosis in LREs.

### Improved protein uptake and survival in *pllp* mutants is gradual and recurrent

To test whether we could recover the impaired LRE uptake phenotype observed in the original pre-adapted *pd1116*-/- fish, we outcrossed our current *pd1116*-/- stock to the WT AB strain (*pd1116*-AB+/-) which carries different genetic background but shows similar protein absorption capability to EK (Fig 6A, 6C) [49]. We performed a one generation outcross of *pd1116*-/-to AB to test if we could segregate the increased protein uptake phenotype (Fig 6A). A *pd1116*-/-parent whose offspring from a sibling in-cross showed enhanced mTurquoise absorption at 30 min post-gavage than EK or AB (Fig 6C) was outcrossed to an AB parent to generate F1 *pd1116*-AB+/-. We then in-crossed the F1 *pd1116*-AB+/- adults and assayed 3 different clutches of mixed larvae followed by genotyping. Similar to previous results, we observed no significant differences in mTurquoise signal level at 30 min post-gavage among *pd1116*-AB+/+, +/- and -/- offspring across all 3 different pairs of F1 parents we tested (S5D Fig). We continued with outcrossing *pd1116*-/- for 4 consecutive generations to AB, and in F2-F4 *pd1116*-AB+/- tested the offspring of sibling in-crosses for their protein uptake capability. Remarkably, protein absorption levels in the *pd1116*-AB-/-larvae of the tested offspring populations of F2-F4 *pd1116*-AB+/- incross did not revert to the pre-adapted impaired protein uptake phenotype but rather appeared to normalized with comparable uptake level to their *pd1116*-AB+/+ siblings (representative quantification shown in Fig 6B). These results indicate that LRE hyperactivity can be lost by outcrossing the inbred line to a different genetic background (Fig 6D). However, we did not detect a simple pattern of inheritance that could be attributed to one gene modifier.

**Fig 6:**
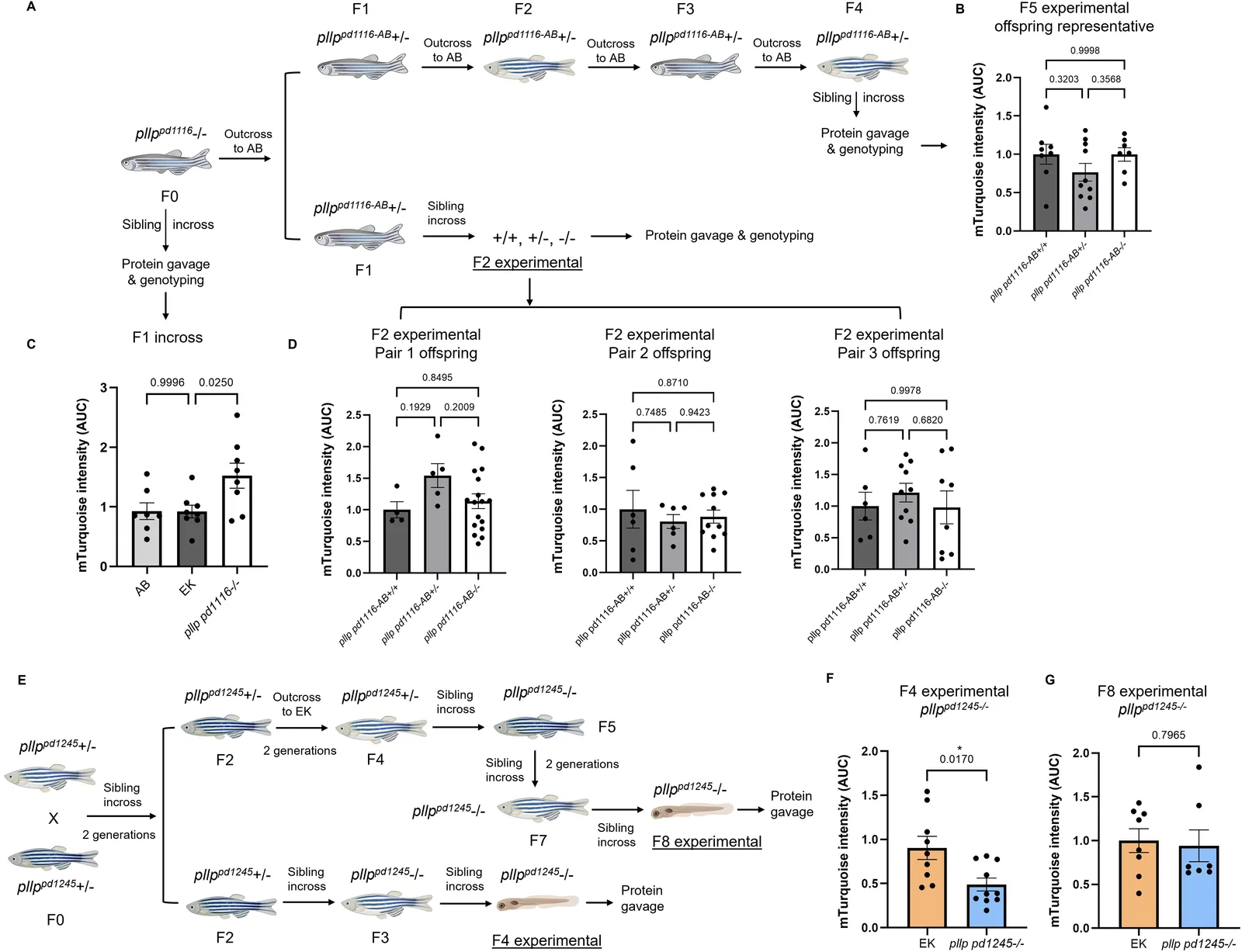
Complex and recurrent adaptation of *pllp* mutant alleles. **(A)** Pedigree of *pd1116-/-* outcrossed to unrelated WT AB; top: 3 pairs of F2 *pd1116-AB*+/- sibling in-crosses were tested; 5 pairs of F3 *pd1116-AB*+/- sibling in-crosses were tested; 6 pairs of F4 *pd1116-AB*+/- sibling in-crosses (F5 experimental larvae) were tested. Bottom: 4 pairs of F1 *pd1116-AB*+/- sibling in-crosses (F2 experimental larvae). Created in BioRender. W, S. (2026) https://BioRender.com/mgzlhdv **(B)** Representative quantification of F5 experimental offspring mTurquoise uptake level at 30min pg (from F4 *pd1116-AB*+*/-* sibling incross) showing normalization of -/- uptake capability to +/+; n = 8 for +/+, n = 10 for +/-, n = 7 for -/-. **(C)** 30min pg mTurquoise protein uptake level comparison between the offspring of EK, AB and the *pd1116-*/- parents used for outcrossing; n = 8 for EK; n = 7 for AB; n = 8 for *pd1116-*/-. **(D)** Quantifications of LRE region mTurquoise protein level at 30 min pg in 4 different clutches of F2 offspring of F1 *pd1116-AB*+/- sibling in-cross; data normalized to *pd1116-AB*+/+; one-way ANOVA; increased uptake of protein in *pd1116-/-* compared to WT is lost in outcrosses but reduced uptake is not recovered even after several outcrosses. **(E)** Pedigree of the *pd1245* allele lineage; loss of reduced mTurquoise uptake level was observed starting at the in-cross experimental offspring of F5 *pd1245-/-* parents (2 in 3 clutches showed normalized protein uptake). Created in BioRender. W, S. (2026) https://BioRender.com/mgzlhdv **(F, G)** Quantifications of LRE region mTuquoise fluorescent level at 30min pg in 6dpf F4 *pd1245-/-* experimental in-cross larvae and 6dpf F8 *pd1245-/-* experimental in-cross larvae; n = 9 for EK, n = 10 for F4 *pd1245-/-* **(F)**; n = 9 for EK, n = 7 for F8 *pd1245-/-.* **(G)**; unpaired t-test; data normalized to EK.

To test whether the adaptation observed in *pd1116*-/- is recurrent, we in-crossed *pd1245*+/- according to the pedigree shown in Fig 6E. Apart from maintaining the genetic line as depicted, we also tested the protein absorption capability of the offspring of sibling in-crosses from each generation (experimental offsprings). Interestingly, we started to notice a normalization to EK levels of protein uptake levels in *pd1245*-/- starting in F6 in-cross experimental offspring (2/3 clutches), with normalization increasing in F7 incross experimental offsprings (4/5 of clutches) and becoming complete in F8 experimental *pd1245*-/- larvae (Fig 6F, 6G). In contrast, the F4 experimental *pd1245*-/- larvae derived from a separately maintained parental line still demonstrated significantly impaired uptake compared to EK (Fig 6F). It is worth noting that during the early maintenance of the *pd1245* allele, the *pd1245* founder heterozygous adults had been in-crossed for 2 generations before they were moved to an outcross scheme for future generations, possibly contributing to the normalization of the *pd1245* allele to EK level in our genetic experiment.

Given that F8 *pd1245*-/- larvae showed protein uptake activity comparable to that of EK (Fig 6F, 6G), we next asked whether this adaptation was accompanied by changes in survival, growth, and immune gene expression. We therefore performed a new 20‑day feeding experiment using the same HP and LP diets, raising EK and F8 *pd1245*-/- larvae from 6 dpf to 20 dpf under the same calorie‑restricted regime used to assay the other alleles (S7A Fig). We found that survival trajectories were broadly similar between EK and F8 *pd1245*-/- under both HP and LP conditions, although EK survival under LP was lower than in the initial experiment shown in Fig 1G, likely reflecting inter‑experimental variation in LP sensitivity rather than a consistent genotype effect (S7B Fig). In contrast to the pre‑adapted F4 *pd1245*-/- cohort, which exhibited significantly reduced standard length relative to EK under both diets (Fig 1H), the adapted F8 *pd1245*-/- larvae displayed standard lengths indistinguishable from EK under HP or LP conditions, consistent with normalization of growth in the adapted line (S7C Fig).

To determine whether the heightened immune gene expression observed in F4 *pd1245*-/- (Fig 5G) was relieved upon adaptation, we performed bulk RNA‑seq at the 20 dpf endpoint. Principal component analysis revealed that both diet and genotype contributed to variance in global expression patterns, with HP and LP samples separating along PC2 and EK versus F8 *pd1245*-/- separated modestly along PC1 (S7D Fig). We then focused on a curated panel of pro‑inflammatory, antimicrobial, immune‑homeostasis, antiviral/defense‑response, and T‑cell‑associated genes previously examined in Fig 5G. Because changes in library preparation and sequencing methods led to unreliably low counts for some genes, we restricted our analysis to genes with robust expression across both the original (EK vs F4 *pd1245*-/-) and new (EK vs F8 *pd1245*-/-) datasets. As reported above, the pre‑adapted F4 *pd1245*-/- cohort showed elevated expression of many pro‑inflammatory and antimicrobial genes relative to EK, particularly under LP conditions (Figs 5G and S7E, left two panels). By contrast, in the adapted F8 *pd1245*-/- line, expression of none of the immune genes we could assess differed significantly from EK under either HP or LP diets (S7E Fig, right two panels). These findings indicate that the multigenerational normalization of protein uptake and growth in *pd1245*-/- is accompanied by a relaxation of the heightened immune gene expression previously observed in the pre‑adapted state, yielding an immune expression profile that resembles that of wild-type EK for the genes examined here.

Altogether, our studies suggest that broad transcriptional changes lead to increased protein absorption and a fine-tuned regulation of immune genes that collectively play a role in the adaptation to the loss of Pllp function, from a protein malnutrition state to one with normalized physiology that is transmitted across generations.

## Discussion

In this study, we demonstrate that loss of function in zebrafish *pllp* leads to severe protein malnutrition due to impaired LRE function, but long-term maintenance of the *pd1116*-/- line in homozygosity results in a robust and heritable functional recovery. Rather than a single discrete mutation, this recovery can be best understood as a process of adaptive transcriptional remodeling under persistent selection, where chronic loss of *pllp* imposes sustained physiological stress that favors regulatory states enhancing nutrient uptake and balancing immune responses over successive generations. Through systematic comparison of the adapted *pd1116*-/- line with a newly generated, non-adapted *pd1245* allele and parental WT EK, we identified two likely adaptive mechanisms: 1) Transcriptional upregulation of the endocytic machinery in LREs, resulting in a hyperactivated protein-absorptive state that even exceeds WT capacity; 2) A diet-dependent remodeling of immune gene expression that simultaneously dampens energetically costly and potentially damaging inflammatory responses while accelerating the maturation of targeted adaptive immunity. While it is not possible to ascertain whether these two mechanisms occurred concurrently or gradually in phases, it seems likely that increased protein uptake is an early event as recent work suggested that transcription of genes encoding the uptake machinery, particularly *cubn* and *dab2* highly sensitive to environmental conditions [30].

Remarkably, the adapted *pd1116*-/- larvae do not merely restore LRE protein absorption to wild-type level but surpass it via the coordinated upregulation of several genes including *transmembrane phosphatase with TEnsin homology (tpte)*, *dab2*, *cubn*, and *amn*, thus reinforcing multiple parts of the endocytic pathway. While these observations support a robust increase in protein uptake and degradation, they do not yet establish whether canonical LRE biology itself has been restored or whether the improvement reflects broader tissue-level compensation. Future work dissecting LRE ultrastructure and single-cell transcriptional states will be required to resolve this distinction. Furthermore, it is notable that the similar active LRE cell numbers among *pd1116*-/-, *pd1245*-/- and EK contrast with what was suggested by earlier, largely qualitative analysis used in Rodríguez-Fraticelli et al. (2015). In this study, we quantified active LRE numbers volumetrically across the intact gut using dextran gavage and segmentation, which is a more complete and quantitative approach than the imaging of gut cross-sections used previously. Similarly, we have now assayed protein uptake quantitatively using a kinetic assay and whole-gut fluorescence signal rather than snapshots from transverse sections, enabling more accurate comparison of total absorptive capacity across genotypes. These methodological improvements provide greater confidence that the enhanced protein absorption phenotype reported for the adapted *pd1116* allele reflects a real increase in protein uptake and degradation. Moreover, the original feeding experiment in Rodríguez-Fraticelli et al. (2015) did not adopt the calorie-restricted HP/LP feeding scheme; instead, larvae were fed a single undefined larval diet which is no longer available. This methodological difference precludes exact comparison of current survival rates to the pre-adapted state. Thus, the apparently attenuated severity of *pd1245* should be interpreted with this caveat in mind.

### Immune remodeling in *pllp* mutant adaptation

The second adaptive pillar involves immune regulation. Immune-related transcriptional changes are among the most intriguing features of the adapted state, but at present, the evidence for a causal role in improved survival is indirect. We therefore regard these changes as a plausible component of the adapted state rather than as a defined driver, and future experiments will be needed to determine their functional contribution. Dissection of our RNAseq data and GO analyses revealed a dampening of pro-inflammatory reactions and an increased T cell function marker activation. The increased T cell activity may be due to an increase in the systemic antigen load experienced by the adapted fish due to increased transcytosis [51,52]. The simultaneous suppression of pro-inflammatory gene expression in *pd1116*-/- are consistent with a shift toward dampened innate inflammation and enhanced adaptive immune engagement. Inflammatory innate immune responses can cause tissue damage to the gut [50] and other tissues, thus tuning down inflammatory innate responses while speeding up the maturation of the more targeted adaptive immune responses may be beneficial in improving survival, especially under nutrient restriction [29–31,37,38,51]. This is illustrated by the pronounced divergence of responses under HP conditions, the dietary context in which LRE hyperactivity would increase transcytosis of luminal protein antigens and bacterial metabolites into other organs via circulation. This also suggests that the predicted decrease in sensitivity to bacteria in *pd1116*-/- from our GO term analysis may represent a protective response. Furthermore, because the endocytic machinery is negatively regulated by microbial signals [30], reduced sensitivity to bacteria in the adapted *pd1116*-/- fish may account for the increased activity compared to WT. However, whether protein uptake in the adapted *pd1116* allele is insensitive to microbial signals is unclear.

The new RNA‑seq analysis of the adapted F8 *pd1245*-/- line further refines our view of how immune remodeling relates to adaptation in *pllp* mutants. In the pre‑adapted F4 *pd1245*-/- cohort, which shows the most severe impairment in protein uptake, survival, and growth, we observed abnormal expressions of multiple pro‑inflammatory and antimicrobial genes relative to EK, particularly under LP conditions. This pattern is consistent with a dysregulated immune state in children experiencing persistent malnutrition [53–55]. In the adapted F8 *pd1245*-/- line, however, as protein uptake and growth have normalized to EK levels, expression patterns of the curated list of immune genes also became comparable to that of EK under both HP and LP diets. These observations suggest that the heightened immune activation seen in severely malnourished *pd1245*-/- larvae is alleviated as survival, growth, and intestinal protein uptake recover, and that in this allele the adapted state converges toward a wild-type‑like immune expression profile. These findings are consistent with a scenario in which the more immuno‑suppressive signature in *pd1116‑/-* is associated with its enhanced protein uptake and transcytosis, whereas the adapted *pd1245*-/- displays WT‑like protein uptake and normalized immune gene expression but does not exhibit a similar secondary immunomodulatory phase. Taken together with the *pd1116‑*/- data, these results support a model in which improved protein uptake and survival in *pllp* mutants are generally accompanied by a normalization in the dysregulated immune state associated with severe protein malnutrition, even though the precise immune endpoint differs between alleles. Because our analyses are based on bulk RNA‑seq and a selected immune‑gene panel and were generated using different library preparations, these conclusions remain correlative and do not exclude more subtle or cell‑type‑specific changes in immune regulation. Future work combining cell‑resolved transcriptomics and functional immune assays will be required to disentangle the contributions of antigen load, barrier integrity, and systemic metabolic state to these outcomes.

### Metabolic rewiring in the adapted state

WGCNA revealed that *pd1116*-/- adaptation is accompanied by metabolic rewiring. The Yellow module, which is constitutively upregulated in the adapted *pd1116*-/-, is enriched not only for endocytic and immune pathways, but also for amino acid catabolism. These pathways eventually feed into the TCA cycle, potentially boosting mitochondrial energy production independently of canonical glucose and amino acid oxidation. This would place additional strain on mitochondrial function, making mitophagy dependent protective pathways crucial [56]. In this context, downregulation of autophagy in *pd1245*-/- may partially explain the loss of LRE activity.

### Genetic origin of nutritional adaptation

While our analyses have uncovered key functional mediators of the adaptation of *pd1116*-/-, the genetic origin remains unclear. Here, we use ‘adaptation’ as a descriptive term to capture the stable, transgenerational divergence in survival, endocytosis, and transcriptional control observed in the in-bred *pd1116*-/- line compared to newly generated alleles. We emphasize that this functional divergence could reflect a range of underlying mechanisms—including the selection of genetic modifiers, accumulation of background variation, epigenetic shifts, or transcriptional adaptation—and its exact molecular basis remains to be fully resolved. Both *pd1116*-/- and *pd1245*-/- transcripts showed nonsense-mediated mRNA decay (NMD), which has been shown to lead to transcriptional adaptation (TA), the compensatory upregulation of sequence-related genes, typically paralogs, via transcriptional effects driven by mRNA decay products [57–61]. This response may also be epigenetically inherited across generations through germline-carried signals, with H3K4me3 enrichment at adapting gene promoters detectable in early embryos of subsequent generations [62,63]. This mechanism is therefore a plausible contributor to the stable, multigenerational transcriptional changes we observe in *pd1116*-/-. However, known *pllp* paralogs including *CKLF-like MARVEL transmembrane domain-containing protein 3* (*cmtm3)*, *cmtm6*, *cmtm7*, *cmtm8a*, *Mal, T cell differentiation protein 2 (mal2)*, *MARVEL domain-containing protein 1 (marveld1)*, *proteolipid protein 2b* (*plp2b)*, and *mal, T cell differentiation protein b (malb)*, did not show significant upregulation in either *pd1116*-/- or *pd1245*-/- compared to EK (S6 Fig), as canonical NMD-triggered TA would predict. *cmtm3* and *cmtm8a* were upregulated in *pd1116*-/- relative to *pd1245*-/- under HP conditions, and *cmtm3* and *cmtm7* showed the same pattern under LP conditions. Another important difference between the adaptation observed in *pd1116*-/- and canonical TA is that while the former seems to arise gradually, the latter occurs acutely within hours of expressing a mutated mRNA subject to NMD [57]. Taken together, these observations suggest that if transcriptional adaptation contributes to the adapted state in *pd1116*-/-, it likely does so in a non-canonical or partial form and is not sufficient on its own to explain the broad, genome-wide changes we observe. Our current data therefore do not allow us to distinguish TA from other mechanisms such as selection of genetic modifiers or epigenetic remodeling. Furthermore, the gradual emergence of improved survival and protein uptake over many generations, combined with the absence of a simple segregation pattern in our outcross experiments, is compatible with a model in which several genetic modifiers of small effect accumulate under strong selection, potentially acting together with transcriptional and epigenetic mechanisms.

Finally, it is worth noting that the *pd1116* allele was generated with a TALEN, while *pd1245* was generated with CRISPR/Cas9. It is possible that these two genome engineering methods alter chromatin organization and gene expression in different ways. Beyond the differences in editing modality, it is also important to recognize that the original *pd1116* mutation could not be precisely recreated due to the technical limitations of the early CRISPR-based approaches used for generating the *pd1245* allele. However, the phenotypes of both alleles are consistent with complete loss of function, as both produce large truncations and also show pronounced *pllp* transcript degradation by RT-PCR, consistent with NMD. Nevertheless, it is possible that differences in the genomic lesion between the two alleles, or subtle differences in the residual mutant RNA fragments produced, contribute to the divergent degrees of phenotypic severity relative to what was originally reported for *pllp* loss of function. These differences in editing modality and lesion context therefore reinforce the need for cautious interpretation of allele-to-allele comparisons.

Although our findings show that a zebrafish intestinal mutant line can undergo substantial phenotypic and transcriptional remodeling under persistent nutritional stress, extrapolation to broader epigenetic or evolutionary mechanisms should be made cautiously. Future work dissecting the relative contributions of genetic modifiers, NMD-linked processes, and epigenetic regulation will be required to place this phenomenon in a wider evolutionary context.

## Materials and methods

### Fish

The Duke University Institutional Animal Care and Use Committee (IACUC) guidelines were followed in the care and use of all fish in this project. We maintained zebrafish (*Danio rerio*) stocks on a recirculating system at 28°C with a controlled, 14-hour light and 10-hour dark cycle. Genotypes were determined by fin clipping. Male and female breeders from 3-9 months of age were used to generate fish for all experiments. Breeding adult zebrafish were fed a 1:1 ratio of GEMMA Micro (Skretting) and artemia. To breed fish, males and females were placed in mating tanks with dividers overnight, and dividers were removed the following morning. 5–30 dpf zebrafish larvae from the Ekkwill (EK) or AB background were used in this study. No test on the influence of sex was performed as sex determination in zebrafish occurs in late juvenile stage, well after our experimental window. Strains generated for this study: *pllp*^*pd1116*^*, pllp*^*pd1245*^*, pllp*^*pd1309*^.

### Genome editing

Mutant lines and CRISPR-Cas9 pool knockout fish were generated using CRISPR/Cas9. Guide RNA (gRNA) target sites were identified using CRISPRscan [64] and gRNAs were synthesized using the oligo-based method [65]. *pllp pd1245* mutants were generated using a gRNA targeting exon2, and *pllp pd1309* mutants also at exon2. gRNA target sequences were: *pd1245*–5’ GGTTGCTGAGTCTTCATCAG 3’, and pd1309-5’ GGTTCTGAGCATTGCCCTGC 3’. gRNA target sequences for *pllp* CRISPR-Cas9 pool are: 5’ GGCTGAGTGCTGACCTTCCC 3’, 5’ GGTTCTGAGCATTGCCCTGC 3’, 5’ GGACCCTGGTAATACCCACC 3’, 5’ GGTGAAGGGCAGAACGCAGC 3’. gRNA target sequences for *slc45a2* CRISPR-Cas9 pool are: 5’ GGACTACTGTAGGTCGTCAT 3’, 5’ GGGTCCGTCAATGAAGTCTG 3’, 5’ GGATGCTAACCATAGCTGAC 3’, 5’ GGCTATGCCACCTATGAGAG 3’. Zebrafish embryos were injected at the one cell stage with 150 pg/nl of Cas9 mRNA and 50 pg/nl of gRNA.

Genotyping for *pd1116* was performed using primers: forward, 5’ GCACTCAGACCAGCTCACAA 3’; (DCAPS primer [66]) reverse, 5’ CGGAACAGAAAAGTGGGTGT 3’. PCR products were then digested with BsmI enzyme for genotyping. Genotyping for *pd1245* was performed using primers: forward, 5’ GCTGGTTATCCTGTTGCTGAT 3’; reverse, 5’ GCATTAGCCAGAAAGGCAGT 3’. Genotyping for pd1309 was performed using primers: forward, 5’ GATTATTAGTGTGGACGCTGATTGC 3’; reverse, 5’ AATGTTCCCTCCTTCATGAATAGTC 3’.

### RNA isolation and reverse transcription PCR (RT-PCR)

RNA was extracted using the RNeasy Mini Kit (Qiagen) according to the manufacturer’s protocol. cDNA was synthesized using first Strand cDNA Synthesis Kit (Roche). To generate cDNA for RT-PCR, poly dT primer was used for reverse transcription cDNA synthesis. Primers used for RT-PCR are: pllp_F 5’ GGCGGATTTTCCTGGGAAGG 3’, pllp_R 5’ CCGTGCTCCCTGCTGC 3’; rpl13a_F 5’ TCTGGAGGACTGTAAGAGGTATGC 3’, rpl13a_R 5’ AGACGCACAATCTTGAGAGCAG 3’. *rpl13a* was used as standard.

### Fluorescent protein purification

Previously described methods were used to prepare mCherry and mTurquoise [3].

### Microscopy

Whole-mount confocal live or fixed sample imaging was performed on a 1) Leica SP8 confocal microscope with a 25X objective (Leica) and (Leica), or 2) Olympus confocal microscope with a 30x/1.05 silicone oil objective (Olympus) and FluoView software (Olympus). Fish were mounted onto glass-bottom dishes in a 0.9% agarose mixture of egg water and 1x tricaine. Z-step size for z-stacks images was 1.5 μm.

### Gavage assays

Larvae were sedated with 0.22 µm-filtered 1X Tricaine (0.2 mg/mL). Sedated larvae were suspended in 3% methyl cellulose and gavaged with ~4 nL of fluorescent cargo [67]. Larvae were placed in a 28°C zebrafish incubator to absorb the fluorescent cargo following gavage.

### mTurquoise uptake & degradation

Larvae were gavaged with mTurquoise (2.5 mg/mL) and incubated for 30min. Luminal mTurquoise was cleared with a 1X PBS gavage. Following clearance, larvae were preserved in 4% PFA in PBS immediately following clearance, 30min post clearance or 60min post clearance. Samples were stored at 4° C overnight. The following day, larvae were mounted on glass bottomed dishes and imaged by confocal microscopy. Fluorescence profiles of mTurquoise signal in LREs were generated by analyzing confocal images in ImageJ (version 1.53t). Images were z-projected as max intensity plots. The LRE region was delineated with a rectangular selection tool to generate plot profiles of mTurquoise fluorescence along the LRE region. Signal intensity was calculated from area under the curve (AUC) measurements in PRISM Graphpad. The average AUC measure at each time point was calculated and plotted. These values were normalized by dividing them by the average AUC value at 30 minutes post gavage in EK.

The rate of mTurquoise degradation was calculated by doing a simple linear regression on the plotted average AUC values at 30/60/90min pg. The differences in the degradation slopes among EK and pllp mutants or CRISPR knock-out groups were calculated and compared for differences in mTurquoise degradation rates.

### mCherry trans-epithelial transport

6dpf EK*, pllp* ^*pd1116*^*-/-,* and *pllp* ^*pd1245*^*-/-* larvae were sedated & gavaged with mCherry protein (1.25 mg/mL) and incubated for 4 hours. After that, sedated larvae were mounted on glass-bottom plates and live imaged by confocal microscopy. The average mCherry intensity in the pronephros organ was quantified and compared using 1-way ANOVA with Tukey’s multiple comparisons test.

### Dextran568 gavage for LRE segmentation

6dpf EK*, pllp* ^*pd1116*^*-/-,* and *pllp* ^*pd1245*^*-/-* larvae were sedated & gavaged with a dextran568-phenol red gavage mixture (1.5mg/mL Dextran568 in 1X PBS with 0.03% phenol red) using the same method described previously. After 1 hour of incubation, the larval gut was cleared as described above.

### LRE segmentation

Following dextran568 gavage, larvae were stored in the zebrafish incubator for 26 hours. LRE segmentation was done as previously described in Childers et al., 2025. Statistics were done by 1-way ANOVA with Tukey’s multiple comparisons test.

### *In situ* hybridization chain reaction (HCR)

HCR was done as previously described in Childers et al., 2025 [68]. Briefly, larvae were imaged by confocal microscopy using the Olympus system. Fluorescence profiles of each sample were quantified by taking the ratio of the average signal of the LRE region divided by the average signal of the background area. The differences in quantified fluorescence profiles among larvae being compared were calculated with 1-way ANOVA with Tukey’s multiple comparisons test.

### Zebrafish growth and survival under controlled high- and low-protein diet feeding conditions

Sibling larvae from EK, *pllp*^*pd1116*^-/-, and *pllp*^*pd1245*^-/- incrosses were conventionally raised from 0-5 dpf. At 6 dpf, 10 larvae/3L tank were placed on our recirculating Aquatic Habitats system. From 6-20 dpf, larvae were fed 10 mg/day of a custom formulated high (HP) or low-protein (LP) diet daily between 11 am-12 pm. Diet formulations are described in detail below. The survival of fish was monitored up to 20 dpf. Then, surviving fish were collected and imaged at 20 dpf with an Axio Zoom.V16 Stereoscope (Zeiss) with ZEN pro 2012 software (Zeiss). Using the acquired images, fish standard lengths [69] were measured with ImageJ. A separate image of 10mm ruler under the same zoom parameter was taken as the size reference.

### Diet formulations

Previously described diet formulations were used in the above feeding experiments [30].

### RNA extraction and bulk RNA sequencing and analysis

At the end of feeding assay (Fig 1G), 3 out of 4–5 tanks per each genotype/diet group were randomly selected as biological replicates. All surviving zebrafish larvae within each tank were pooled for RNA extraction, yielding one pooled sample per replicate. This resulted in 3 biological replicates per condition across 6 conditions (3 genotypes x 2 diets), for a total of 18 samples submitted for bulk RNA sequencing.

For RNA extraction, zebrafish larvae were collected, rinsed in ultra-pure water, and immediately snap-frozen in liquid nitrogen. Tissues were homogenized into a fine powder using a pre-chilled, RNase-free disposable pellet pestle. Total RNA was extracted by adding 300 µL of TRIzol reagent and allowing the homogenate to thaw. Following the addition of 60 µL of chloroform and vigorous vortexing, the phases were separated using Phase Lock Gel Heavy tubes (QuantaBio) centrifuged at 12,000 rcf for 15 minutes at 4°C.

The aqueous phase was recovered and further purified using the RNeasy MinElute Cleanup protocol (Qiagen). Briefly, the sample was mixed with 70% ethanol and transferred to an RNeasy MinElute spin column. The membrane was washed with Buffer RPE and 80% ethanol, followed by a 5-minute centrifugal drying step with open lids to eliminate residual ethanol. RNA was eluted in 14 µL of RNase-free water. RNA concentration was evaluated by Qubit (Thermo Fisher Scientific) and integrity was assessed using an Agilent 2100 Bioanalyzer. Samples with an RNA integrity number (RIN) >7.0 were used for sequencing. Libraries were prepared using a stranded mRNA-Seq protocol and sequenced on the Illumina NovaSeq 6000 platform (S-Prime flow cell) with 50 bp paired-end reads.

Raw sequence quality was assessed using FastQC (v0.11.7) [70], and adapter sequences and low-quality bases were removed using Trim Galore (v0.6.5) [71]. Trimmed reads were aligned to the Zebrafish genome (GRCz11/danRer11) and the corresponding Ensembl transcriptome [72] (Release 100) using STAR (v2.7.3a) [73]. Genome indices were generated using a --sjdbOverhang of 49. For alignment, --outFilterMultimapNmax was set to 1 to retain only uniquely mapped reads, and --outSAMstrandField intronMotif was utilized for downstream compatibility.

Gene quantification was performed using HTSeq (v0.11.1) [74]. Differential expression analysis was conducted in R using DESeq2 (v1.26.0) [75]. Prior to normalization, genes were retained if they had at least 10 reads in at least 3 samples; all others were removed prior to DESeq2 normalization (32,520–22,863 genes). The DESeq function was used to normalize data and estimate dispersions. Differential expression was modeled using the Wald test. Two design formulas were used on the filtered counts. A full model, ~ genotype + diet + genotype:diet, was used for the variance-stabilizing transformation (the basis for PCA and expression heatmaps) and to test genotype × diet interaction terms. An equivalent group model, ~ condition (where each condition is a genotype–diet combination), was used to extract all pairwise contrasts — genotypic differences within each diet and dietary effects within each genotype — by the Wald test.

Genes were considered differentially expressed if they exhibited a |log2 fold change| > 1 and an adjusted p-value < 0.05 (Benjamini-Hochberg correction [76]). For visualization, Variance Stabilizing Transformation (VST) was applied to the count data. Principal Component Analysis (PCA) was performed on VST-transformed data using DESeq2 (plotPCA) and visualized with the ggplot2 package.

Functional enrichment analysis was performed on the DEGs identified from all experimental comparisons, including dietary effects within each genotype (HP vs. LP for EK, 1116, and 1245) and genotypic differences under specific diets (1116 vs. EK and 1245 vs. EK for both HP and LP conditions). Over-representation analysis for Gene Ontology (GO) Biological Process (BP) and KEGG pathways was conducted using the clusterProfiler package. Redundant GO terms were collapsed with *simplify(cutoff = 0.6)* and the ten most significant terms per gene set are plotted in Fig 5A and 5B. To maintain statistical rigor, custom backgrounds were utilized for all tests, consisting of all genes detected in the RNA-seq dataset (22,863 genes for GO using gene symbols and 20,439 genes for KEGG using Entrez IDs). P-values were adjusted using the Benjamini-Hochberg method, with a significance threshold of adjusted p < 0.05.

For S7 Fig, bulk RNA was extracted as above from EK and pd1245 larvae (HP and LP diets; 14 samples). Library preparation, sequencing, and primary data analysis were performed by Plasmidsaurus: reads were quality-filtered with fastp v0.24.0, aligned to GRCz11 with STAR v2.7.11, UMI-deduplicated (UMIcollapse v1.1.0), and quantified with featureCounts (subread v2.1.1; strand-specific, fractional multimapping, grouped by gene_id); Plasmidsaurus differential expression used edgeR/TMM. For the immune-gene comparison, gene-level counts from this dataset were re-analyzed in DESeq2 using the same design (~ condition for the pd1245-vs-EK contrast within each diet; ~ genotype + diet + genotype:diet for the variance-stabilizing transformation) to match the primary pipeline (genes with ≥10 counts in ≥3 samples retained; Wald test for pd1245 vs EK within each diet; variance-stabilizing transformation for the z-scored heatmap). No curated immune gene reached padj < 0.05 for pd1245 vs EK in either diet.

### Weighted gene co-expression network analysis (WGCNA)

Weighted gene co-expression network analysis was performed using the WGCNA package [32] in R to identify modules of highly co-expressed genes. To focus the analysis on biologically informative variation, count data were first normalized using a Variance Stabilizing Transformation (VST) from the DESeq2 package [75]. The top 25% most highly variable genes (based on variance) were retained for network construction, resulting in a dataset of 5,716 genes.

A signed hybrid co-expression network was constructed using a soft-thresholding power (β) of 18. Modules were identified using the blockwiseModules function with a maximum block size of 20,000 genes, a minimum module size of 30, and a merge cut height of 0.25. For each module, a Module Eigengene (ME)—representing the first principal component of the expression profile—was calculated. Module-trait relationships were evaluated by correlating MEs with experimental traits using Pearson correlation. To visualize expression patterns across genotypes and diets, module eigengene values were plotted as boxplots.Functional annotation was performed using the clusterProfiler [77] package and the org.Dr.eg.db zebrafish database. Gene Ontology (GO) enrichment analysis for biological processes (BP) and KEGG pathway enrichment were conducted for genes within specific modules. Enrichment analysis was performed using the standard organism-wide background to ensure broad capture of functional categories. P-values were adjusted for multiple testing using the Benjamini-Hochberg (BH) method, with a BH-adjusted p < 0.05.

### Ethics statement

Animal husbandry and experimental procedures followed the guidelines set forth by the Association for Assessment and Accreditation of Laboratory Animal Care (AAALAC). All zebrafish experiments were performed in accordance with protocols approved by the Duke University Institutional Animal Care and Use Committee (IACUC) under protocol registry number 57 approved on February 21st, 2026.

## Supporting information

S1 Table Differentially expressed genes (DEGs) across genotypes and dietary conditions in zebrafish larvae. Lists of DEGs identified via pairwise differential expression analysis using DESeq2, factoring in both diet and genotype as covariates. DEGs were strictly defined by a False Discovery Rate (FDR; padj) < 0.05 and an absolute Log2fold change (|LFC|) > 1. The spreadsheet is organized into nine distinct tabs corresponding to each pairwise comparison: Tab 1 (pd1116 HP vs LP): DEGs reflecting the dietary effect within the pd1116-/- mutant genotype. Tab 2 (pd1245 HP vs LP): DEGs reflecting the dietary effect within the pd1245-/- mutant genotype. Tab 3 (EK HP vs LP): DEGs reflecting the dietary effect within the wild-type EK genotype. Tab 4 (HP 1116 vs EK): DEGs showing genotype differences between pd1116-/- mutants and EK wild-type under High Protein conditions. Tab 5 (HP 1245 vs 1116): DEGs showing genotype differences between pd1245-/- and pd1116-/- mutants under High Protein conditions. Tab 6 (HP 1245 vs EK): DEGs showing genotype differences between pd1245-/- mutants and EK wild-type under High Protein conditions. Tab 7 (LP 1116 vs EK): DEGs showing genotype differences between pd1116-/- mutants and EK wild-type under Low Protein conditions. Tab 8 (LP 1245 vs 1116): DEGs showing genotype differences between pd1245-/- and pd1116-/- mutants under Low Protein conditions. Tab 9 (LP 1245 vs EK): DEGs showing genotype differences between pd1245-/- mutants and EK wild-type under Low Protein conditions. Each tab contains gene symbols, base mean expressions, Log2 fold changes, standard errors, test statistics, p-values, and adjusted p-values.
(XLSX)

S2 Table Genotype-specific differentially expressed genes (DEGs) under High Protein and Low Protein conditions. Lists of DEGs derived from cross-genotype comparisons that highlight distinct, non-overlapping transcriptional responses relative to the wild-type EK line. DEGs were identified using DESeq2 (padj < 0.05 and |LFC| > 1). The spreadsheet is organized into four distinct tabs: Tab 1 (genes_unique_to_1116_HP_table): Mutant-specific DEGs unique to the pd1116-/- line under High Protein (HP) conditions, containing genes that are significantly altered in the pd1116-/- vs. EK comparison but do not overlap with the pd1245-/- vs. EK dataset. Tab 2 (genes_unique_to_1116_LP_table): Mutant-specific DEGs unique to the pd1116-/- line under Low Protein (LP) malnutrition conditions, containing genes that are significantly altered in the pd1116-/- vs. EK comparison but do not overlap with the pd1245-/- vs. EK dataset. Tab 3 (genes_unique_to_1245_HP_table): Mutant-specific DEGs unique to the pd1245-/- line under High Protein (HP) conditions, containing genes that are significantly altered in the pd1245-/- vs. EK comparison but do not overlap with the pd1116-/- vs. EK dataset. Tab 4 (genes_unique_to_1245_LP_table): Mutant-specific DEGs unique to the pd1245-/- line under Low Protein (LP) malnutrition conditions, containing genes that are significantly altered in the pd1245-/- vs. EK comparison but do not overlap with the pd1116-/- vs. EK dataset. Each tab provides standard differential expression outputs, including gene symbols, base mean expressions, Log2 fold changes, standard errors, test statistics, p-values, and adjusted p-values. These partitioned lists correspond to the distinct genotype-specific sectors visualized in the Venn diagrams of S2B-S2C Fig.
(XLSX)

S1 Fig Analyses of pllp locus sequence and mRNA splicing. (A, B) DNA sequencing results and predicted amino acid sequences at the original *pd1116*-/- mutation site (A) and the *pd1245*-/- mutation site (B); numbers indicate bp downstream of the ATG start codon. (B) Predicted protein sequences of Pllp *pd1116*-/- (top) and Pllp *pd1245*-/- (bottom); numbers indicate aa downstream of the Met start site. (D) bigwig file of the pllp locus showing tracks for EK, *pd1116*-/- and *pd1245*-/- under both diet conditions. (E) IGV visualization of splice junction coverage at the pllp locus for EK and *pd1116*-/- under both diet conditions; no novel splicing patterns are detected. (F) DEX Seq analysis of relative exon usage at the *pllp* locus. (C, C’) RT-PCR of *pllp* and *rpl13a* control (C) and quantifications of band intensity (C’) comparing EK, *pd1116*-/- and *pd1245*-/-. Both mutant transcripts undergo non-sense mediated decay; n = 3 for each genotype.
(TIF)

S2 Fig Transcriptomic profiling reveals genotype-dependent and diet-dependent gene expression programs in *pllp* mutants. (A) Venn diagram showing overlap of diet-responsive DEGs (HP vs. LP) among EK, *pd1116*-/-, and *pd1245*-/-, illustrating genotype-specific transcriptional sensitivity to diet. (B) Venn diagram of DEGs identified in HP *pd1116*-/- vs. EK and HP *pd1245*-/- vs. EK, highlighting genes uniquely dysregulated in each mutant under HP conditions. (C) Venn diagram of DEGs identified in LP *pd1116*-/- vs. EK and LP *pd1245*-/- vs. EK, revealing the expanded set of *pd1245*-unique DEGs under nutritional stress. See also S2 Table. (D) Eigengene expression box plots for the 12 co-expression modules (grey, unassigned genes, excluded) across all experimental groups (EK-HP, EK-LP, *pd1116*-HP, *pd1116*-LP, *pd1245*-HP, *pd1245*-LP), illustrating the genotype and diet specificity of each module. Yellow and Green modules show strong *pd1116*-specific patterns independent of diet, while Black and Turquoise modules show combined genotype and diet effects. Boxes show the median and interquartile range of the 3 replicates per group, whiskers extend to 1.5 × IQR, and outliers are omitted. Box fill indicates the module colour.
(TIF)

S3 Fig KEGG pathway analysis shows *pd1245* mutation disrupts lipid metabolism, extracellular matrix integrity, and cell cycle progression. (A) Comparative KEGG pathway enrichment (compareCluster) of DEGs uniquely dysregulated in *pd1245*-/- relative to EK (non-overlapping with *pd1116*-/- vs. EK DEGs). Four gene lists were analyzed (left to right): upregulated and downregulated DEGs unique to *pd1245*-/- under HP and LP conditions, respectively. Dot size indicates gene count; color indicates adjusted p-value. (B) Heatmap of gene expression across all 18 samples, annotated by genotype and diet for selected functional categories: lipid/cholesterol metabolism and vitamin digestion and absorption (including the components of LRE endocytic machinery cubn and amn). Color scale represents Z-score. In *pd1245*-/-, these genes are broadly downregulated, with the most severe suppression under LP conditions. In *pd1116*-/-, expression is largely normalized, particularly under HP conditions, demonstrating transcriptional compensation of these critical pathways.
(TIF)

S4 Fig Normal LRE numbers and largely unaffected expression of genes characteristic for various intestinal cell populations in *pd1116*-/- and *pd1245*-/-. (A) Gene expression heatmap of 43 intestinal cell-type markers for the absorptive enterocytes, LREs, enteroendocrine cells, goblet cells and mucins. Heatmap plotted according to per-gene z-scores of variance-stabilized counts across all 18 samples, averaged within each genotype × diet group. Markers as in Fig 5G (* FDR < 0.05 EK vs *pd1116*; # FDR < 0.05 *pd1116* vs *pd1245*, within diet). (B) Experimental setup of active LRE quantification at 6dpf using dextran568 and using Ilastik object segmentation. Created in BioRender. W, S. (2026) https://BioRender.com/mgzlhdv. (C) Representative images of the LRE regions showing dextran568-filled LRE lysosomal vacuoles. (D) Quantifications of active LRE cell numbers; n = 8 for EK, n = 9 for F7 *pd1116*-/-, n = 6 for F5 *pd1245*-/-; one-way ANOVA.
(TIF)

S5 Fig pllp CRISPR gRNA pool efficiency test, *slc45a2* CRISPR gRNA pool as a valid knock-out control and characterization of the *pd1309* allele. (A) Diagram of the *pllp pd1309* allele generated by CRISPR-Cas9 single gRNA; editing results in a 21 bp in-frame deletion within TM2. (B) DNA sequencing result (top) and predicted protein sequence (bottom) of the *pllp pd1309* allele compared to EK; numbers indicate bp downstream of the ATG start codon. (C, C’) RT-PCR of *pllp* and *rpl13a* control (C) and quantifications of band intensity (C’) comparing EK and *pd1309*-/-; *pd1309*-/- does not demonstrate *pllp* transcript decay; n = 3 for each genotype. (D, E) mTurquoise fluorescent signal profile (D) and quantifications (E) at 30min pg; n = 4 for F2 *pd1309* + / + , n = 11 for F2 *pd1309* + /-, n = 5 for F2 *pd1309*-/-; data normalized to F2 *pd1309* + / + ; one-way ANOVA. (F) Percentage of GFP + TgBAC(*pllppd1114*-GFP)+/- x EK larvae either noninjected (NIC) or injected with the pllp CRISPR gRNA pool (EK+ *pllp* pool). Loss of GFP indicates that knockdown of *pllp* was efficient. (G) DIC images of EK and EK + *slc45a2* CRISPR gRNA pool; *slc45a2* knockout results in near complete loss of skin pigments. (H) Quantifications of body lengths of EK NIC and EK injected with *slc45a2* CRISPR gRNA pool. (I, J) Fluorescent profile (I) and quantifications (J) of mTurquoise signal at 30min pg; n = 7 for EK NIC; n = 8 for EK + *slc45a2* pool; upaired t-test.
(TIF)

S6 Fig *pllp* paralog expression heatmap. *pllp* sequence match indicates the % of paralog sequences matching *pllp* sequence. No significant upregulation of any paralogs in *pd1116*-/- compared to EK. Data from ensembl.org.
(TIF)

S7 Fig Feeding experiment and bulk RNAseq results comparing EK and F8 *pd1245*-/-. (A) Schematics of feeding experiment with calorie-restricted high-protein diet (HP) and calorie-restricted low-protein (LP) diet. (B) Survival curves of EK and F8 *pd1245*-/- over 20 days under HP or LP diet. n = 4 tanks/condition for EK and n = 5 tanks/condition for *pd1245*-/-, starting 10 fish/tank. (C) Standard lengths of surviving fish of 2 genotypes at 20dpf. n = 32 for EK HP; n = 41 for F8 *pd1245*-/- HP; n = 22 for EK LP; n = 25 for F8 *pd1245*-/- LP. One-way ANOVA. (D) Principal component analysis (PCA) of global gene expression. PC1 separates genotypes, while PC2 separates diets. EK and *pd1245*-/- shows clear separation by genotype along PC1. Both genotypes show effects from diet-dependent separations. (E) Immune panel heatmaps of selected immune genes from Fig 5G showing comparisons between EK and F4 *pd1245*-/- (left two panels) and comparisons between Ek and F8 *pd1245*-/- (right two panels). Note that due to changes in library preparation and sequencing methods, some genes have unreliably low counts and therefore are excluded from E. No genes exhibit significant changes comparing EK to F8 *pd1245*-/- in E. Heatmap plotted according to raw z-scores.
(TIF)

S1 Raw Gel DNA gels were imaged with BioRad ChemiDoc MP Imaging System equipped with Image Lab5.2 software. (PDF)
