# Supplementary material for: Genome wide transcriptional changes underlie gradual and recurrent adaptation to protein malnutrition in zebrafish
Source: PLoS Genet. 2026 Sep 28;22(9):e1012071. doi: 10.1371/journal.pgen.1012071 (PMC13652339; doi:10.1371/journal.pgen.1012071)
Supplement: Supplementary file 1 [file pgen.1012071.s010.pdf]

Figure S1C raw gel image

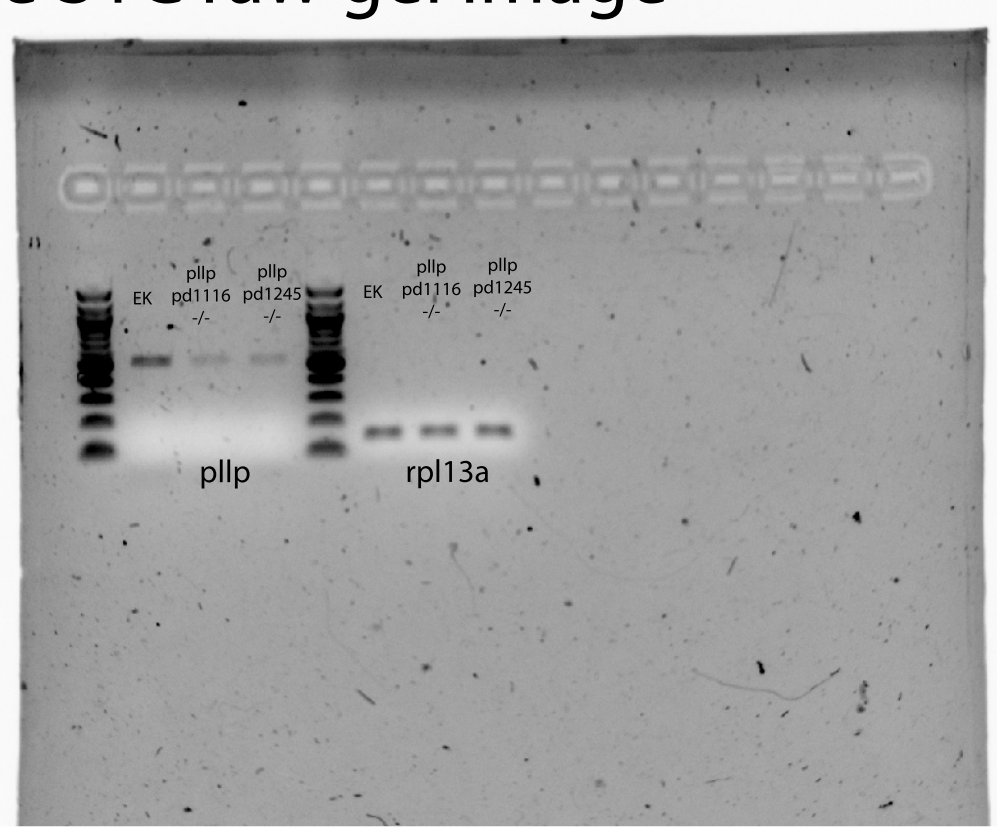

Figure S5C raw gel image

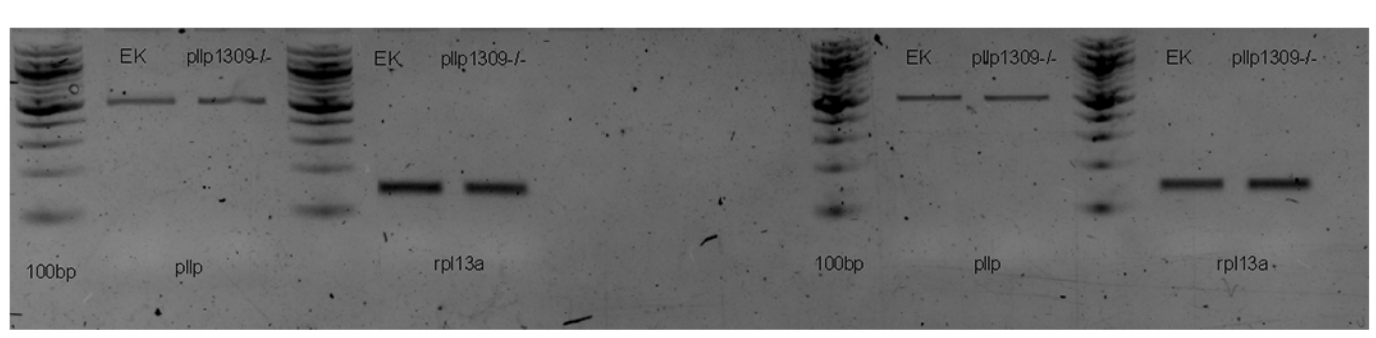

DNA gels were imaged with BioRad ChemiDoc MP Imaging System equiped with Image Lab5.2 software.
