# Supplementary material for: Genome wide transcriptional changes underlie gradual and recurrent adaptation to protein malnutrition in zebrafish
Source: PLoS Genet. 2026 Sep 28;22(9):e1012071. doi: 10.1371/journal.pgen.1012071 (PMC13652339; doi:10.1371/journal.pgen.1012071)
Supplement: Supplementary file 2 [file pgen.1012071.s012.docx]

We thank the reviewers for their kind and positive assessment of our manuscript and for their insightful comments and constructive criticism. We have followed their suggestions and perform an RNAseq study to address a major experimental question and also performed an additional experiment to document the effect of a mutation on the transcript. We have also followed their suggestions to make editorial changes to the text. Below we provide point -by-point detailed answers to all issues raised by the reviewers.

**Comments to the Authors:
Please note here if the review is uploaded as an attachment.**

**Reviewer #1**: Summary: Loss of plasmolipin (pllp) has previously been shown to reduce zebrafish survival due to impaired LRE development and dietary protein absorption. Acute and generational adaptation to nutritional stress, such as a low protein diet, has previously been demonstrated. How an organism may adapt to a heritable defect in protein absorption is less clear. In this study, Wang et al. demonstrate generational adaptation to pllp loss of function through transcriptional remodeling that increases protein uptake. In parallel, they observe remodeled expression of immune response genes, and postulate that this immune remodeling compensates for an increased antigen exposure. They demonstrate that the uptake adaptation is recurrent in a second more recently generated pllp allele. The authors are quite thorough in generating multiple alleles to support their hypothesis, which was noted and appreciated by this reviewer. These findings would be of interest to the readers of PLoS Genetics. We recommend the following revisions to strengthen the conclusions and improve the clarity of the manuscript.

We suggest the following critiques be addressed prior to publication:
1. The authors show that in two independent pllp alleles, protein uptake defects can be recovered after an adaptation period. In the pd1116 background, the adaptation leads to enhanced protein uptake and an immunosuppressive phenotype is also observed. Does this phenotype also develop in the adapted pd1245 background? This could be assessed through either transcriptomics or targeted quantification in the adapted pd1245 background of a subset of differentially expressed immune genes that emerged in the pd1116 transcriptional dataset. If adapted pd1245 are immuno-wild-type, this would strengthen the authors hypothesis that immunosuppression is a reaction to increased antigen load that the adapted pd1245 do not experience because they have wild-type protein uptake. This would further suggest that the adaptation in pd1116 may have occurred in two phases - the first being increased protein uptake, the second being immunomodulation, a phase that pd1245 has not yet encountered.

We thank the reviewer for raising this issue. As suggested, we performed a new HP/LP feeding experiment using wild-type EK and an adapted F8 *pd1245*-/- line in which intestinal protein uptake is comparable to EK, followed by bulk RNA‑seq. We specifically assessed the subset of immune genes that were differentially expressed in the initial *pd1116* dataset, using only genes with robust counts across both experiments. This limitation was due to the need to use a lower-coverage, more affordable, platform for the latest RNAseq experiment.

In contrast to the pre‑adapted F4 *pd1245*-/- cohort, which showed elevated expression of multiple pro‑inflammatory and antimicrobial genes relative to EK, we no longer detected significant differences between EK and F8 *pd1245*-/- for any of the measured immune genes under either HP or LP diets, and survival and growth were also indistinguishable between genotypes in this assay. Within the sensitivity of our analysis, the adapted *pd1245*-/- line, therefore, appears close to wild‑type for this immune gene panel. These findings are consistent with the reviewer’s hypothesis that the more immuno‑suppressive signature in pd1116‑/- is associated with its enhanced protein uptake and transcytosis, whereas adapted *pd1245*-/- with WT‑like uptake does not exhibit a similar secondary immunomodulatory phase. We have added these data and their interpretation to the Results (final subsection) and Supplementary Figure S7 in the revised manuscript.

While we cannot say definitely whether increased protein uptake and the modulation of immune responses occurred concurrently or sequentially, it is likely that, as suggested by the reviewers, this is a gradual event that happened in phases. In support of this interpretation, we also note that based on recent work we cite, transcription of the uptake machinery genes seems highly sensitive to environmental conditions.

2. The authors produce a pllp LOF allele pd1309 that carries a 21-bp in-frame deletion, and like the other unadapted alleles, demonstrates a protein uptake deficiency. While the other alleles undergo NMD, the authors suggest that this allele does not, but the transcriptional proof is not reported. Please provide either additional RT-PCR or qPCR data to support this statement.

We have added a representative RT-PCR gel image and quantification results of band intensities in Figure S5C & C’. This semi-quantative RT-PCR was done using 3 replicates for each genotype, and statistical comparison didn’t point to significant differences in *pllp/rpl13a* transcript ratio between EK and *pllp pd1309-/-*, suggesting that the allele is subject to NMD.

Minor issues
1. In the first paragraph of the results section, it would be beneficial to the reader to include that the pllppd1116 mutant was generated through TALEN-mediated mutation harboring an indel that leads to a frame shift and premature termination codon. This information was not readily available in the methods or fully described in the text.

Thank you for pointing this out. We have clarified in the text that *pllp^pd1116^* mutant is generated through TALEN-mediated nonsense mutation in *pllp*.

“Previously, in Rodríguez-Fraticelli et al. (2015) we reported that zebrafish with a **TALEN-generated nonsense mutation** in *pllp* (expression shown in Figure 1A), named *pllp^pd1116^-/-,* showed severely impaired survival performance when raised with a limited food supply (AP100 commercial zebrafish diet; 80-90% reduction in survival rate), and exhibited dramatically reduced endocytosis of fluorescently labeled dextran in LREs.”

2. The presentation of the survival curves in Figure 1 and the description in the text was confusing. It would have been helpful to see the original survival graph from the 2015 paper in order to compare to the survival curves on the defined diets in 2015 and 2021. If the defined diets were present in 2015 (Fig 1C), then why did the original paper use the undefined diet? Some of this is noted in the discussion, but it would be helpful to include more details in the results section.

To align with citation and reproduction guidelines, we decided to cite the previously published data instead of redrawing the original survival graph in this manuscript. However, to facilitate comparison, we added the percentage reduction in survival in the text.

Regarding the diet question: in the original paper, the published feeding experiment was done by our collaborators in a different facility in Spain in 2014 at a time when the defined diets were not yet available, and so a commercial (undefined) diet was used instead. When the defined High Protein (HP) and Low Protein (LP) diets subsequently became available, we re-tested the survival experiment under these controlled dietary conditions to better model a protein-deficient challenge. To clarify this progression for readers, we have added further detail to the manuscript explaining the transition from the commercial diet used in 2015 to the HP/LP diets used in the later experiments.

3. In Figure 1E, please provide labeling of where the exons are located. The text describes that certain exons were targeted for mutation and it would be nice to see the exons in relation to the TL and TM domains. This also applies to Figure S5F.

We have modified the graphs for both figures to include exons aligned to domains.

4. The figure panels are mislabeled in Figure S1 – current panels C and C’ are F and F’ in the legend and in the manuscript text. This alters the labeling for current panels D, E and F.

Thank you for pointing this out. We have made the corrections.

5. Please provide more information about the RNAseq samples. There is no mention of how many samples were run and how many fish were in each sample. Please include this both in the methods and in the main text (or at least in the figure legend).

Thank you for this suggestion. We have added clarification regarding the RNA-seq sample number and pooling strategy in both the main text and the methods section.

Briefly, for each of the 6 conditions (3 genotypes × 2 diets), 3 tanks were randomly selected from 4–5 available tanks as independent biological replicates. All surviving larvae within each tank were pooled for RNA extraction, yielding one pooled sample per replicate and a total of 18 samples for bulk RNA sequencing. The number of surviving fish per tank varied by genotype and diet condition, which is reflected in the survival data shown in Figure 1G. These details have now been incorporated into the methods section and in the relevant results section.

6. On line 137, the results start discussing IGV and DEXseq analysis, which the reviewer assumes to have come from the RNAseq dataset? At this point in the results, the RNAseq has not been introduced yet, so it is not clear whether that assumption is correct.

We thank the reviewer for the helpful suggestion. We agree that introducing these analyses prior to describing the RNA-seq dataset created confusion regarding the source of the data. We have revised the manuscript to describe the RNAseq dataset first, and now discuss the IGV and DEXsesq analyses immediately following that introduction. This restructuring clarifies where the data originated and improves the overall narrative flow of the results section.

“(After describing RNAseq dataset; specifically hierarchical clustering of all expressed genes) To investigate whether alternative splicing or restored transcript variants contributed to *pd1116-/-*’s transcriptomic recovery, we analyzed exon-level usage from our RNA-seq dataset. Integrative Genomics Viewer (IGV) visualization of splice junction coverage combined with Differential Exon Usage Sequence (DEXseq) analysis of relative exon usage at the *pllp* locus revealed the absence of novel splice isoforms or exon skipping, ruling out alternative splicing as a driver of *pd1116*-/-’s improved survival (Figure S1D-F).”

7. Please justify further in the text why the pllp crispant data was included and not just the data from the new stable pd1309 allele.

We have included in the text justifying the use of *pllp* CRISPR pool and rearranged some paragraphs to improve the flow. More specifically, we moved the pd1309 allele paragraph before the CRISPR pool so that the alleles are grouped together and that the CRISPR pool serves as an acute test that further validates consequences of *pllp* loss-of-function on top of the two independent alleles.

8. In Figure 3, it would be helpful to see example images of the HCR fluorescence for dab2, cubn and amn expression in each of the mutants, not just in the EK wild-type background. The corresponding brightfield images do not need to be included. Further, please add labels to the axes in C’, D’ and E’.

We have replaced the HCR images with representative images from the 3 genotypes.

Thank you for pointing out the formatting errors. We have added the corresponding labels.

9. In Figure S5C, the Y axis is only from 3.4 to 4.2 mm, such that it makes it appear that the fish exhibit a much larger difference in length (despite being statistically non-significant). Please use the full 0 to 4.2 mm axis.

Thank you for pointing this out. We have made the corrections.

10. In Figure 6A, the schematic could be simplified by eliminating the lower F1 section and including the sibling in-cross etc. notation directly under the F1 on the top row. This would be much easier for the reader. Additionally, if you are not showing the F3 assay data, it would be best not to include the sibling in-cross etc. notation below the F3 generation. It may also help the reader to re-organize the manuscript text related to this figure to talk about the F1 generation first before discussing the assay effect seen in the F5 generation.

We have simplified the graphs as suggested. We have also reorganized text paragraphs to first discuss F1 outcross results.

11. Please use the same scale on the Y axes in Figure 6D for easier comparison between graphs.

Thank you for pointing this out. We have made the corrections.

12. In Figure 6F & G, make sure the figure panel, legend and manuscript text are all referring to the same F4 and F8 generation.

Thank you for pointing this out. We have made the corrections.

13. When possible, we recommend using the whole phrase for infrequently used acronyms like post-gavage (PG) and non-injected control (NIC) in the main body of the manuscript.

Thank you for pointing this out. We have made the corrections.

14. It would help the reader to note in the figure legends the generation number (F#) for each allele that is shown in a given figure panel so it is clear when the authors are showing data from adapted or non-adapted alleles.

We added in the figure legends the generation number (also shown below) as suggested.

Figure 1C: pd1116 F2

Figure 1D: pd1116 F6

Figure 1F-H: pd1116 F6, pd1245 F4

Figure 2 pd1116 F6, pd1245 F4Figure 3: pd1116 F7, pd1245 F5

Figure S4: pd1116 F7, pd1245 F5

Figure 4: pd1116 F6, pd1245 F4

Figure 5: pd1116 F7, pd1245 F5

Figure S5: pd1309 F2

15. Occasional spelling errors were noted, such as line 9 (Carolina), line 301 (phosphatase)

Thank you for pointing this out. We have made the corrections.

16. There are a number of places where it seems that reference information is missing – see line 506, 645

Thank you for bringing this to our attention. We have corrected these instances in the revised manuscript:

**Line 506:** “We maintained zebrafish (Danio rerio) stocks on a recirculating system at 28°C with a controlled, 14-hour light and 10-hour dark cycle **()**.”

- Removed the empty parentheses, as this sentence describes standard zebrafish husbandry conditions and does not require a reference citation.

**Line 645:** “The DESeq**()** function was used to normalize data and estimate dispersions.”

- Removed the empty parentheses following DESeq. The reference for DESeq is cited earlier in the Methods section where the package is first introduced.

**Reviewer #2**: This manuscript examines how zebrafish can adapt over generations to the loss of pllp, a gene required for the differentiation and function of lysosome-rich enterocytes. Earlier work had already shown that pllp mutants have impaired intestinal protein uptake, reduced growth, and poor survival under protein-poor conditions. The main advance of the present study is the analysis of a mutant line that appears to have partially overcome this defect after long-term maintenance, together with a comparison to a newly generated allele that still shows the original phenotype. The authors combine physiological assays and transcriptomic analysis to argue that this adaptation is associated with improved absorptive function in the intestine and broader changes in epithelial and immune-related programs. This is an interesting and potentially important story. At the same time, some of the stronger claims, particularly those concerning the basis of the adaptation and the weight given to some mechanistic interpretations, would benefit from a more careful framing.
Overall, I find the study interesting and suitable for Plos Genetics, as it addresses a relevant question using a well-chosen model and provides a useful set of observations. The main issues are not so much about missing large experimental blocks, but rather about how some of the conclusions are presented and interpreted. In particular, the claims around adaptation and mechanism should be toned down, and a few points would benefit from clearer validation or more cautious wording. With these revisions, I think the manuscript would be significantly strengthened without requiring extensive additional work.

Major points

1. What exactly is meant by “adaptation”? The most important claim in the paper is that the long-maintained mutant line has adapted in a way that improves intestinal function and survival. The phenotypic difference between the older line and the newly generated allele is interesting and, in several respects, quite convincing. Still, I think the paper goes a bit too far in how it interprets that difference.

What the data clearly show is that the two mutant lines are not equivalent anymore. What is not yet established is the basis of that divergence. At the moment, this could reflect selection of genetic modifiers, background effects accumulated over time, or some other stable change associated with long-term maintenance. The manuscript often treats this as an established genetic adaptation, but that seems stronger than the data currently support.

I would strongly suggest toning down the wording throughout. This does not necessarily require a large new experimental effort, but the text should separate more clearly what is directly shown from what remains an interpretation.

We thank the reviewer for this important conceptual point. The data clearly demonstrate that the adapted *pd1116-/-* line and the newly generated *pd1245-/-* line are no longer phenotypically equivalent, but the basis of this divergence — whether genetic modifiers, background drift, epigenetic changes, or other stable alterations — remains to be determined.

We have revised the manuscript throughout to replace causal or mechanistic language with observational language, and we have added an explicit statement in the discussion (copied below) clarifying that we use "adaptation" as a descriptive term for the stable phenotypic and transcriptional divergence observed in the long-maintained *pd1116*-/- line, and explicitly acknowledging that its underlying genetic basis remains unresolved. We believe these changes more accurately describe what the data show while preserving the biological significance of the observed divergence.

“While our analyses have uncovered key functional mediators of the adaptation of *pd1116-/-*, the precise genetic origin remains unclear. Here, we use 'adaptation' as a descriptive term to capture the stable, transgenerational divergence in survival, endocytosis, and transcriptional control observed in the long-maintained *pd1116-/-*, line compared to newly generated alleles. We emphasize that this functional divergence could reflect a range of underlying mechanisms—including the selection of genetic modifiers, accumulation of background variation, epigenetic shifts, or transcriptional adaptation—and its exact molecular basis remains to be fully resolved.”

2. Transcriptomic changes are interesting, but still mostly descriptive. The RNA-seq data are useful and add an important layer to the study. They show that the adapted line differs broadly from the non-adapted mutant, including changes in epithelial and immune-related programs. That said, the manuscript sometimes reads as though these pathways explain the adaptive phenotype, when in fact the data are still largely correlative.

I think this is mostly a matter of presentation. The transcriptomic analysis supports the idea that the adapted state is biologically distinct, but it does not really establish which changes are causal and which are secondary. Some sections of the Results and Discussion should be written a bit more cautiously. In particular, the authors should avoid giving the impression that they have identified the mechanism of adaptation unless they can support that more directly.

We thank the reviewer for this important suggestion. Beyond our previous adjustments, we have further revised the WGCNA and metabolic rewiring sections to make the correlative nature of these analyses explicit. We added a sentence stating that co-expression modules capture correlation rather than direct regulation and should be viewed as candidate transcriptional programs. These changes are intended to align the text more closely with the descriptive and hypothesis-generating scope of the transcriptomic analyses.

“Because co-expression modules capture correlation rather than direct regulation, these associations are interpreted as candidate transcriptional programs linked to the adapted state rather than as established causal pathways.”

3. The interpretation of improved LRE function should be more careful. The paper argues that the adapted mutants show improved absorptive function, and this is one of the strongest parts of the story. Still, I was not always convinced that the data distinguish clearly between restoration of canonical LRE function and more general compensation at the tissue level.

This matters because the conclusions sometimes imply that LRE biology itself has been restored. From the data as presented, it seems safer to say that absorptive performance is improved, but that the precise basis of that improvement is still not fully resolved. If the authors want to keep a stronger statement here, they should justify it more explicitly. Otherwise, a small adjustment in wording would probably be enough.

Thank you for the suggestion. In addition to the wording changes mentioned above, we have now revised the end of the LRE results section (copied below) to emphasize that upregulation of endocytic machinery genes is closely associated with, rather than stating it confers, higher protein absorption in the long-maintained *pd1116*-/- line. In the discussion section, we explicitly state that our current data do not resolve whether canonical LRE function is restored or whether the improvement reflects more general tissue-level compensation, and we frame this as an important question for future work.

“Together, these data show that transcriptional upregulations of key LRE endocytic machinery genes, rather than changes in LRE numbers, is closely associated with the higher protein absorption capacity observed in the long-maintained *pd1116*-/- line, although the precise cellular mechanisms underlying this improvement remain to be defined.”

4. Comparison with the newly generated allele is valuable, but the limits of the comparison should be acknowledged. Using a newly generated pllp allele as a reference is a strong part of the paper. It gives the whole story much more weight, because it provides a plausible representation of the original mutant phenotype before long-term adaptation or drift.

At the same time, the manuscript could be more explicit about the limits of this comparison. The two lines are being used to support fairly strong conclusions, but it is hard to know how much of the difference reflects adaptation in the older line versus other background-related variables. I am not saying the comparison is invalid — only that the interpretation should be a bit more measured. A short discussion of this issue in the text would help.

Thank you for the suggestion. In the discussion section, we have integrated the differences in editing modality between the TALEN-generated *pd1116* allele and the CRISPR/Cas9-generated *pd1245* allele into our conceptual framing, noting that these differences are an additional reason to interpret allele comparisons cautiously.

5. Some of the broader claims should be scaled back. The paper is at its strongest when it stays close to the actual system: a zebrafish intestinal mutant line that appears to have changed over time in a reproducible and biologically meaningful way. Where it becomes less convincing is when the discussion broadens into more general claims about adaptive mechanisms or evolutionary implications.

I would recommend keeping the conclusions closer to the data. The study already has a solid and interesting story without needing to overextend its scope. In my view, a more restrained discussion would actually improve the paper and make the main findings easier to appreciate.

In line with the reviewer’s suggestion, we have trimmed and reframed the more speculative portions of the text and added cautionary discussion languages. We also added a concise statement at the end of the discussion, emphasizing that extrapolation should be cautious and that further work will be needed to dissect the underlying genetic and epigenetic mechanisms.

Minor points

1. Some conclusions read more strongly than the data justify. In a few places, especially in the Results, the wording is a bit too definitive. This is most noticeable when the manuscript moves from describing a phenotypic difference to assigning a mechanism. I think the paper would benefit from slightly more restrained language in those sections. The story is still interesting without pushing every observation to its strongest possible interpretation.

Thank you for bringing this up. We have toned down our languages throughout the text.

2. The distinction between “adapted” and “non-adapted” lines should be handled more carefully in the text. The terminology is understandable, but after a while, it starts to sound as though the nature of the difference has already been settled. Since the basis of that difference is still part of the interpretation, it may be better to remind the reader from time to time that these are operational terms rather than fully established categories. A small adjustment in wording would help avoid overstatement.

Thank you for raising this point. We have adjusted our language regarding adaptation throughout the main text as mentioned in the major point #1.

3. The transcriptomic section is harder to follow than it needs to be. The RNA-seq analysis contains useful information, but the way it is presented feels somewhat dense. It would help to guide the reader more clearly through the logic of the analysis: what was compared, what came out of those comparisons, and which changes the authors consider most relevant to the phenotype. Right now, some of the interpretation arrives a bit too quickly.

We thank the reviewer for this helpful feedback. We have substantially revised the transcriptomic results section to improve clarity and logical flow.

For the DESeq2 analysis, we added an introductory paragraph explicitly stating the model design (*~ genotype + diet + genotype:diet*), the structure of all nine pairwise comparisons (diet effect within each genotype, and genotype effects within each diet condition), and the thresholds used to define DEGs (FDR < 0.05 and |log₂ fold change| > 1). This scaffolding allows the reader to understand the analytical logic before encountering the results of each comparison.

For the WGCNA analysis, we expanded the introductory text to clarify the purpose of the analysis, the network construction approach (signed hybrid network across all 18 samples), and how module eigengenes were correlated with traits. We also explicitly state upfront which of the 12 modules showed significant and biologically interpretable associations with genotype and/or diet, prioritizing the reader's attention on the most relevant findings before describing each module in detail.

Finally, we streamlined the KEGG pathway discussion by removing interpretations not directly supported by our data and retaining only pathways with meaningful associations within each module, reducing speculative language and improving readability throughout.

4. Immune-related changes are interesting, but the discussion could be more balanced. The immune signature is one of the more intriguing aspects of the paper, but at present, it is difficult to know how central it is to the phenotype. The manuscript sometimes gives the impression that these changes may be mechanistically important, but the evidence here is still fairly indirect. I would suggest discussing this as a plausible component of the adapted state rather than as a defined driver.

We have further toned down our interpretation of immune remodeling. In the revised manuscript, we now describe immune changes as a plausible component of the adapted state and explicitly note that the evidence for a causal role in survival is indirect. We removed or softened language that previously implied that immune remodeling was a defined driver of adaptation and clarified that functional validation will be required to establish its precise contribution.

5. A few figure legends could be more explicit. In several places, I had to go back and check exactly what was being quantified and from which samples. This is not a major issue, but the figure legends could do a better job of stating the experimental comparison more directly. It would also help if the legends made clear when results come from biological replicates, pooled samples, or representative images.

We’ve updated our legends of the following figures for improved clarity.

1H: Data per genotype per diet come from pooled larvae from all tanks within that condition

2A: Data for each genotype/diet condition come from pooled larvae from 3 randomly selected tanks within that condition.

3C-E’: Representative images of one selected fish from the indicated genotype/diet condition from one representative experiment are shown here. Signal profile and expression level quantification of HCR in situ hybridization for dab2 (C-C’’), cubn (D-D’’) and amn (E-E’’) in 6dpf EK, F7 pd1116-/-, and F5 pd1245-/- are from one representative out of three experimental replicates that showed similar data trends.

S4D: Quantifications of active LRE cell numbers; data shown are from one representative experiment out of 2 replicates.

4C-E: Data from one representative experiment out of four replicates that showed similar trends.

4F-H: data from one representative experiment out of three replicates that showed similar trends.

S5D, E: Data from one representative experiment out of four replicates that showed similar trends.

S5F-J: Data from one representative experiment out of two replicates that showed similar trends.

5E, F: Data from one representative experiment out of two replicates that showed similar trends.

6G: data from one representative experiment out of four replicates

**Reviewer #3**: This manuscript examines how zebrafish carrying loss-of-function mutations in pllp recover survival and intestinal protein uptake over multiple generations despite persistence of the original deleterious lesion. Using comparisons between an adapted historical allele (pd1116) and a more recently generated non-adapted allele (pd1245), the authors combine survival studies, RNA-seq, co-expression network analysis, and functional uptake assays to argue that adaptation is associated with increased expression of lysosome-rich enterocyte (LRE) endocytic machinery and coordinated immune remodeling. Overall, this is a compelling and technically strong study addressing an interesting biological question. The central observations are convincing and supported by multiple orthogonal approaches. Specific suggestions are provided below.

1. The manuscript convincingly demonstrates recovery of survival and growth in the adapted pd1116 line, increased expression of LRE endocytic machinery, enhanced protein uptake and degradation and coordinated transcriptional changes associated with this phenotype. However, the mechanistic basis of this adaptation remains unresolved. The discussion currently intermixes observed transcriptional remodeling and speculative mechanisms (NMD-driven transcriptional adaptation, epigenetic inheritance). These should be more clearly separated. (e.g. the data support a stable adapted transcriptional state, while the molecular mechanism underlying its emergence and inheritance remains unknown.) Ideas should be clearly framed as hypotheses to avoid over-interpretation relative to the data.

Thank you for this suggestion. We have expanded the "Genetic origin" section to more fully address the reviewers’ comments. Specifically, we now (i) clarify that our data do not provide direct evidence for canonical transcriptional adaptation (TA) and that TA, if present, is likely non-canonical and insufficient on its own to explain the phenotype, and (ii) discuss explicitly the possibility that several genetic modifiers of small effect contribute to the gradual multigenerational trajectory.

2. The use of WGCNA is appropriate and identifies modules associated regulatory programs without additional validation of genotype and diet. However, these modules are correlative and should not be interpreted as causal. Statements implying that modules represent causal regulatory programs could be softened (i.e. it might be more accurate to describe them as coordinated transcriptional programs associated with the adapted state.)

We thank the reviewer for this important clarification. We agree that WGCNA identifies correlative rather than causal relationships, and that our original language overstated the mechanistic implications of module associations.

In response, we have revised the description of all WGCNA modules to use language that reflects the correlative nature of the analysis as mentioned in our response to the reviewer 2 major point #2. Specifically, we replaced phrases implying causal regulatory programs with more conservative language describing coordinated transcriptional programs associated with each genotype or diet condition. We also substantially shortened the KEGG pathway discussion for each module, removing speculative interpretations not directly supported by our findings and retaining only the most robustly enriched pathways. For example, the Yellow module description was condensed to focus on the primary enriched pathways (cytoskeletal organization, endocytosis, NOD-like receptor signaling, and amino acid metabolism), and the concluding interpretation was reframed to describe association rather than causation.

3. The recovery of survival across generations could plausibly reflect selection of several genetic modifiers of small effect during inbreeding, rather than (or in addition to) transcriptional reprogramming. The authors note that the crosses reported in Figure 6D do not support the presence of a single genetic modifier, but they should consider in the discussion the possibility of multiple genetic modifiers, particularly given the multigenerational timescale, the gradual emergence of the phenotype and the absence of direct evidence for epigenetic inheritance.

We agree with this point and now explicitly consider multiple modifiers in the Discussion. We state that the gradual, multigenerational emergence of improved survival and protein uptake, together with the lack of a simple segregation pattern in our outcrosses, is consistent with selection of several genetic modifiers of small effect that collectively shape the adapted transcriptional state. We also note that our current data do not yet distinguish among contributions from genetic modifiers, transcriptional adaptation, and epigenetic mechanisms.

“Furthermore, the gradual emergence of improved survival and protein uptake over many generations, combined with the absence of a simple segregation pattern in our outcross experiments, is compatible with a model in which several genetic modifiers of small effect accumulate under strong selection, potentially acting together with transcriptional and epigenetic mechanisms”

“Although our findings show that a zebrafish intestinal mutant line can undergo substantial phenotypic and transcriptional remodeling under persistent nutritional stress, extrapolation to broader epigenetic or evolutionary mechanisms should be made cautiously. Future work dissecting the relative contributions of genetic modifiers, NMD-linked processes, and epigenetic regulation will be required to place this phenomenon in a wider evolutionary context.”

4. The authors could consider describing the pllp adaptation as a process of adaptive transcriptional remodeling under persistent selection. In this view, loss of pllp imposes chronic physiological stress, and over multiple generations, individuals with regulatory states that enhance protein uptake and balance immune responses are favored. This results in a stable lineage characterized by coordinated transcriptional changes in endocytic, metabolic, and immune pathways. The molecular basis of this stability (genetic, epigenetic, or both) remains unresolved and should be presented as an open question.

We thank the reviewer for this insightful perspective of our findings. We agree that framing the *pllp* adaptation as a process of adaptive transcriptional remodeling under persistent selection provides a compelling conceptual framework.

In response to this suggestion, we have revised the opening sentences of the discussion section (copied below) to incorporate this framework. Specifically, we now highlight how chronic physiological stress from the loss of *pllp* favors regulatory states that enhance protein uptake and balance immune responses over generations, leading to coordinated shifts across endocytic, metabolic, and immune pathways. Furthermore, we explicitly present the precise molecular basis of this multigenerational stability—whether mediated by genetic modifiers, epigenetic mechanisms, or an interplay of both—as an important open question for future study.

“In this study, we demonstrate that loss of function in zebrafish *pllp* leads to severe protein malnutrition due to impaired LRE function, but long-term maintenance of the *pd1116*-/- line results in a robust and heritable functional recovery. Rather than a single discrete mutation, this recovery can be best understood as a process of adaptive transcriptional remodeling under persistent selection, where chronic loss of *pllp* imposes sustained physiological stress that favors regulatory states enhancing nutrient uptake and balancing immune responses over successive generations. (Sentences describing two adaptive mechanisms) While these findings establish adaptive transcriptional remodeling as the primary driver of phenotypic recovery, the precise molecular basis of this multigenerational stability—whether mediated by genetic modifiers, epigenetic mechanisms, or an interplay of both—remains an important open question for future study.”

Minor suggestions
5. The main text describes 6/13 co-expression modules as ‘significant and biologically interpretable associations’ [203-204], yet Fig 2D shows 8/13 color-coded modules as significant in at least one category. A brief explanation of why 2/8 significant co-expression modules did not contribute biologically interpretable associations would be helpful.

We thank the reviewer for catching this inconsistency. The discrepancy between the text and figure arose from two distinct issues, which we have now addressed.

First, the grey module — which contains genes unassigned to any co-expression module — was inadvertently included in the original Figure 2D heatmap. Although it showed statistically significant trait correlations with two genotypes, it does not represent a biologically coherent module and should not have been included. We have removed it from the updated heatmap.

Second, the greenyellow module showed a statistically significant association with genotype pd1116-/-, but examination of the eigengene boxplots (Figure S2D) revealed an inconsistent pattern: the diet effect on eigengene expression was opposing across genotypes, and the overall genotype effect was modest. We therefore did not consider it readily biologically interpretable and had excluded it from our original summary statement.

To resolve the inconsistency and avoid confusion, we have revised the main text to report all modules with statistically significant trait associations (7 modules, excluding grey), and we now describe our biological interpretation of each of these 7 modules in the results section.

6. Use ‘suggests’ rather than ‘indicates’ or ‘implies’ for hypothesis generating observations derived from co-expression network analysis (e.g. [232-234] “enrichment for PPAR signaling , retinol metabolism , and xenobiotic metabolism indicates that pd1116-/- has selectively suppressed certain detoxification and lipid-sensing programs.”)

We thank the reviewer for this suggestion. We agree that language should reflect the hypothesis-generating nature of co-expression network analysis, and we have systematically replaced "indicates" and "implies" with "suggests" or similar hedged language throughout the WGCNA results section.

For example, the Green module description was revised from *"enrichment for PPAR signaling, retinol metabolism, and xenobiotic metabolism* ***indicates*** *that pd1116-/- has selectively suppressed certain detoxification and lipid-sensing programs"* to *"this* ***suggests*** *that pd1116-/-* ***may have selectively suppressed*** *certain detoxification and lipid-sensing programs."*

Similarly, the Yellow module concluding statement was revised to frame the interpretation as an association rather than a direct claim: *"these pathways suggest that the pd1116-/- enhanced survival phenotype* ***may be associated with*** *cytoskeletal reinforcement, enhanced endocytosis, activation of innate immune responses, and upregulation of metabolic programs."*

7. It should be clarified that the finding that the pd1116 allele confers a higher protein absorption capacity is only correlated with transcriptional upregulation of endocytic machinery genes, albeit supported by LRE numbers. [308-312] “We found that pd1116-/- was able to both take up significantly more mTurquoise protein cargo within 30 min post-gavage (PG) and degrade them much faster than EK or pd1245-/- over a 60 min period (Figure 4B-E). Together, these data show that transcriptional upregulations of key LRE endocytic machinery genes rather than changes in LRE numbers confer a higher protein absorption capacity to the adapted pd1116 allele.”

We thank the reviewer for this comment. We have revised our interpretation of the LRE phenotype in both Results (copied below) and Discussion sections. For details, please refer to our response to Reviewer 2 major point 3 that raises a similar concern.

“Together, these data show that transcriptional upregulations of key LRE endocytic machinery genes, rather than changes in LRE numbers, **is closely associated** with the higher protein absorption capacity observed in the long-maintained *pd1116*-/- line, **although the precise cellular mechanisms underlying this improvement remain to be defined.**”

8. Using full gene names, rather than abbreviations, would improve clarity for readers unfamiliar with these specific genes. Additionally, gene lists associated with specific processes should be supported with appropriate citations.

Thank you for pointing this out. We’ve added full gene names to each gene’s first occurrence.

9. Mean bars of the ‘black’ module in Fig S2 should be colored white (or similar) for visibility.

We thank the reviewer for this observation. We have updated the bar plot for the black module in Figure S2 to display the mean bar in white for improved visibility.
