# Supplementary material for: Genome wide transcriptional changes underlie gradual and recurrent adaptation to protein malnutrition in zebrafish
Source: PLoS Genet. 2026 Sep 28;22(9):e1012071. doi: 10.1371/journal.pgen.1012071 (PMC13652339; doi:10.1371/journal.pgen.1012071)
Supplement: Supplementary file 3 [file pgen.1012071.s013.docx]

We thank the reviewers for their kind and positive assessment of our manuscript. We have followed the suggestions suggestions from Reviewer 2 to make editorial changes to the text.

**Comments to the Authors:**

1. A few statements in the Abstract still sound somewhat more causal than the revised Discussion. For example, “resulting in a capacity for protein absorption that exceeds that of WT”, “is aided by a fine-tuned transcriptional regulation of immune genes”, and the final statement that genome-wide transcriptional changes “underlie” the stable improvements could be softened slightly. I would suggest wording such as “associated with” or “accompanied by”, which would be more consistent with the careful interpretation adopted elsewhere in the revised manuscript.

Following the Reviewer’s suggestions, we have revised the wording in the abstract mentioned above to:

Line 32: “associated with”

Line 33: “accompanied”

Line 38: “associated with”

2. Similarly, at the beginning of the Discussion, the authors refer to “two intertwined adaptive mechanisms”. This still feels a little stronger than the evidence, particularly for the immune component, which is later—and appropriately—described as having an indirect relationship with improved survival. I would simply align the wording in this opening paragraph with the more cautious language used later in the Discussion.

Following the Reviewer’s suggestions, we have revised the wording in the discussion mentioned above to:

Line 513: “likely”

3. There is a small typo in the Results: “fine-fined tuned regulation” should be corrected.

Done. Thank you.
