# Supplementary material for: Genome wide transcriptional changes underlie gradual and recurrent adaptation to protein malnutrition in zebrafish
Source: PLoS Genet. 2026 Sep 28;22(9):e1012071. doi: 10.1371/journal.pgen.1012071 (PMC13652339; doi:10.1371/journal.pgen.1012071)
Supplement: Peer review history

# Peer review: Decision Letter 0

**DOI:** 10.1371/journal.pgen.1012071.r001

**Authors:** Mary Mullins, Fengwei Yu

14 Apr 2026

PGENETICS-D-26-00186

Genome wide transcriptional changes underlie gradual and recurrent adaptation to protein malnutrition in zebrafish

PLOS Genetics

Dear Michel,

Thank you for submitting your manuscript to PLOS Genetics. As you will see, while the reviewers and editors are very positive about the intriguing adaptation phenotype of the pllp mutant that your group identified, there is a consensus that the conclusions need to be qualified as cause and consequence is not yet established definitively. The reviewers have many other helpful comments to improve the manuscript as well. Therefore, we invite you to submit a revised version of the manuscript that addresses the points raised during the review process.

Please submit your revised manuscript by Jun 13 2026 11:59PM. If you will need more time than this to complete your revisions, please reply to this message or contact the journal office at plosgenetics@plos.org. When you're ready to submit your revision, log on to https://www.editorialmanager.com/pgenetics/ and select the 'Submissions Needing Revision' folder to locate your manuscript file.

Please include the following items when submitting your revised manuscript:

* A letter that responds to each point raised by the editor and reviewer(s). You should upload this letter as a separate file labeled 'Response to Reviewers'. This file does not need to include responses to any formatting updates and technical items listed in the 'Journal Requirements' section below.

* A marked-up copy of your manuscript that highlights changes made to the original version. You should upload this as a separate file labeled 'Revised Manuscript with Track Changes'.

* An unmarked version of your revised paper without tracked changes. You should upload this as a separate file labeled 'Manuscript'.

If you would like to make changes to your financial disclosure, competing interests statement, or data availability statement, please make these updates within the submission form at the time of resubmission. Guidelines for resubmitting your figure files are available below the reviewer comments at the end of this letter.

We look forward to receiving your revised manuscript.

Kind regards,

Mary

Mary C. Mullins

Academic Editor

PLOS Genetics

Fengwei Yu

Section Editor

PLOS Genetics

Aimée Dudley

Editor-in-Chief

PLOS Genetics

Anne Goriely

Editor-in-Chief

PLOS Genetics

**Journal Requirements:**

1) Please ensure that the CRediT author contributions listed for every co-author are completed accurately and in full.

At this stage, the following Authors/Authors require contributions: Siyao Wang, Laura Childers, Fernando Martinez III, Michel Bagnat, and Jieun Park. Please ensure that the full contributions of each author are acknowledged in the "Add/Edit/Remove Authors" section of our submission form.

The list of CRediT author contributions may be found here: https://journals.plos.org/plosgenetics/s/authorship#loc-author-contributions

2) We ask that a manuscript source file is provided at Revision. Please upload your manuscript file as a .doc, .docx, .rtf or .tex. If you are providing a .tex file, please upload it under the item type u2018LaTeX Source Fileu2019 and leave your .pdf version as the item type u2018Manuscriptu2019.

3) Please upload all main figures as separate Figure files in .tif or .eps format. For more information about how to convert and format your figure files please see our guidelines:

https://journals.plos.org/plosgenetics/s/figures

4) We have noticed that you have uploaded Supporting Information files, but you have not included a list of legends. Please add a full list of legends for your Supporting Information files after the references list.

5) We notice that your supplementary Figures are included in the manuscript file. Please remove them and upload them with the file type 'Supporting Information'. Please ensure that each Supporting Information file has a legend listed in the manuscript after the references list.

6) Please amend your detailed Financial Disclosure statement. This is published with the article. It must therefore be completed in full sentences and contain the exact wording you wish to be published.

- State what role the funders took in the study. If the funders had no role in your study, please state: "The funders had no role in study design, data collection and analysis, decision to publish, or preparation of the manuscript.".

If you did not receive any funding for this study, please simply state: u201cThe authors received no specific funding for this work.u201d

7) Figures 1A, 5C, and 6A: Please confirm whether you drew the images / clip-art within the figure panels by hand. If you did not draw the images, please provide (a) a link to the source of the images or icons and their license / terms of use; or (b) written permission from the copyright holder to publish the images or icons under our CC BY 4.0 license. Alternatively, you may replace the images with open source alternatives. See these open source resources you may use to replace images / clip-art:

- https://commons.wikimedia.org

- https://openclipart.org/

**Reviewers' comments:**

Reviewer's Responses to Questions

**Comments to the Authors:**

**Please note here if the review is uploaded as an attachment.**

Reviewer #1: Summary: Loss of plasmolipin (pllp) has previously been shown to reduce zebrafish survival due to impaired LRE development and dietary protein absorption. Acute and generational adaptation to nutritional stress, such as a low protein diet, has previously been demonstrated. How an organism may adapt to a heritable defect in protein absorption is less clear. In this study, Wang et al. demonstrate generational adaptation to pllp loss of function through transcriptional remodeling that increases protein uptake. In parallel, they observe remodeled expression of immune response genes, and postulate that this immune remodeling compensates for an increased antigen exposure. They demonstrate that the uptake adaptation is recurrent in a second more recently generated pllp allele. The authors are quite thorough in generating multiple alleles to support their hypothesis, which was noted and appreciated by this reviewer. These findings would be of interest to the readers of PLoS Genetics. We recommend the following revisions to strengthen the conclusions and improve the clarity of the manuscript.

We suggest the following critiques be addressed prior to publication:

1. The authors show that in two independent pllp alleles, protein uptake defects can be recovered after an adaptation period. In the pd1116 background, the adaptation leads to enhanced protein uptake and an immunosuppressive phenotype is also observed. Does this phenotype also develop in the adapted pd1245 background? This could be assessed through either transcriptomics or targeted quantification in the adapted pd1245 background of a subset of differentially expressed immune genes that emerged in the pd1116 transcriptional dataset. If adapted pd1245 are immuno-wild-type, this would strengthen the authors hypothesis that immunosuppression is a reaction to increased antigen load that the adapted pd1245 do not experience because they have wild-type protein uptake. This would further suggest that the adaptation in pd1116 may have occurred in two phases - the first being increased protein uptake, the second being immunomodulation, a phase that pd1245 has not yet encountered.

2. The authors produce a pllp LOF allele pd1309 that carries a 21-bp in-frame deletion, and like the other unadapted alleles, demonstrates a protein uptake deficiency. While the other alleles undergo NMD, the authors suggest that this allele does not, but the transcriptional proof is not reported. Please provide either additional RT-PCR or qPCR data to support this statement.

Minor issues

1. In the first paragraph of the results section, it would be beneficial to the reader to include that the pllppd1116 mutant was generated through TALEN-mediated mutation harboring an indel that leads to a frame shift and premature termination codon. This information was not readily available in the methods or fully described in the text.

2. The presentation of the survival curves in Figure 1 and the description in the text was confusing. It would have been helpful to see the original survival graph from the 2015 paper in order to compare to the survival curves on the defined diets in 2015 and 2021. If the defined diets were present in 2015 (Fig 1C), then why did the original paper use the undefined diet? Some of this is noted in the discussion, but it would be helpful to include more details in the results section.

3. In Figure 1E, please provide labeling of where the exons are located. The text describes that certain exons were targeted for mutation and it would be nice to see the exons in relation to the TL and TM domains. This also applies to Figure S5F.

4. The figure panels are mislabeled in Figure S1 – current panels C and C’ are F and F’ in the legend and in the manuscript text. This alters the labeling for current panels D, E and F.

5. Please provide more information about the RNAseq samples. There is no mention of how many samples were run and how many fish were in each sample. Please include this both in the methods and in the main text (or at least in the figure legend).

6. On line 137, the results start discussing IGV and DEXseq analysis, which the reviewer assumes to have come from the RNAseq dataset? At this point in the results, the RNAseq has not been introduced yet, so it is not clear whether that assumption is correct.

7. Please justify further in the text why the pllp crispant data was included and not just the data from the new stable pd1309 allele.

8. In Figure 3, it would be helpful to see example images of the HCR fluorescence for dab2, cubn and amn expression in each of the mutants, not just in the EK wild-type background. The corresponding brightfield images do not need to be included. Further, please add labels to the axes in C’, D’ and E’.

9. In Figure S5C, the Y axis is only from 3.4 to 4.2 mm, such that it makes it appear that the fish exhibit a much larger difference in length (despite being statistically non-significant). Please use the full 0 to 4.2 mm axis.

10. In Figure 6A, the schematic could be simplified by eliminating the lower F1 section and including the sibling in-cross etc. notation directly under the F1 on the top row. This would be much easier for the reader. Additionally, if you are not showing the F3 assay data, it would be best not to include the sibling in-cross etc. notation below the F3 generation. It may also help the reader to re-organize the manuscript text related to this figure to talk about the F1 generation first before discussing the assay effect seen in the F5 generation.

11. Please use the same scale on the Y axes in Figure 6D for easier comparison between graphs.

12. In Figure 6F & G, make sure the figure panel, legend and manuscript text are all referring to the same F4 and F8 generation.

13. When possible, we recommend using the whole phrase for infrequently used acronyms like post-gavage (PG) and non-injected control (NIC) in the main body of the manuscript.

14. It would help the reader to note in the figure legends the generation number (F#) for each allele that is shown in a given figure panel so it is clear when the authors are showing data from adapted or non-adapted alleles.

15. Occasional spelling errors were noted, such as line 9 (Carolina), line 301 (phosphatase)

16. There are a number of places where it seems that reference information is missing – see line 506, 645

Reviewer #2: This manuscript examines how zebrafish can adapt over generations to the loss of pllp, a gene required for the differentiation and function of lysosome-rich enterocytes. Earlier work had already shown that pllp mutants have impaired intestinal protein uptake, reduced growth, and poor survival under protein-poor conditions. The main advance of the present study is the analysis of a mutant line that appears to have partially overcome this defect after long-term maintenance, together with a comparison to a newly generated allele that still shows the original phenotype. The authors combine physiological assays and transcriptomic analysis to argue that this adaptation is associated with improved absorptive function in the intestine and broader changes in epithelial and immune-related programs. This is an interesting and potentially important story. At the same time, some of the stronger claims, particularly those concerning the basis of the adaptation and the weight given to some mechanistic interpretations, would benefit from a more careful framing.

Overall, I find the study interesting and suitable for Plos Genetics, as it addresses a relevant question using a well-chosen model and provides a useful set of observations. The main issues are not so much about missing large experimental blocks, but rather about how some of the conclusions are presented and interpreted. In particular, the claims around adaptation and mechanism should be toned down, and a few points would benefit from clearer validation or more cautious wording. With these revisions, I think the manuscript would be significantly strengthened without requiring extensive additional work.

Major points

1. What exactly is meant by “adaptation”? The most important claim in the paper is that the long-maintained mutant line has adapted in a way that improves intestinal function and survival. The phenotypic difference between the older line and the newly generated allele is interesting and, in several respects, quite convincing. Still, I think the paper goes a bit too far in how it interprets that difference.

What the data clearly show is that the two mutant lines are not equivalent anymore. What is not yet established is the basis of that divergence. At the moment, this could reflect selection of genetic modifiers, background effects accumulated over time, or some other stable change associated with long-term maintenance. The manuscript often treats this as an established genetic adaptation, but that seems stronger than the data currently support.

I would strongly suggest toning down the wording throughout. This does not necessarily require a large new experimental effort, but the text should separate more clearly what is directly shown from what remains an interpretation.

2. Transcriptomic changes are interesting, but still mostly descriptive. The RNA-seq data are useful and add an important layer to the study. They show that the adapted line differs broadly from the non-adapted mutant, including changes in epithelial and immune-related programs. That said, the manuscript sometimes reads as though these pathways explain the adaptive phenotype, when in fact the data are still largely correlative.

I think this is mostly a matter of presentation. The transcriptomic analysis supports the idea that the adapted state is biologically distinct, but it does not really establish which changes are causal and which are secondary. Some sections of the Results and Discussion should be written a bit more cautiously. In particular, the authors should avoid giving the impression that they have identified the mechanism of adaptation unless they can support that more directly.

3. The interpretation of improved LRE function should be more careful. The paper argues that the adapted mutants show improved absorptive function, and this is one of the strongest parts of the story. Still, I was not always convinced that the data distinguish clearly between restoration of canonical LRE function and more general compensation at the tissue level.

This matters because the conclusions sometimes imply that LRE biology itself has been restored. From the data as presented, it seems safer to say that absorptive performance is improved, but that the precise basis of that improvement is still not fully resolved. If the authors want to keep a stronger statement here, they should justify it more explicitly. Otherwise, a small adjustment in wording would probably be enough.

4. Comparison with the newly generated allele is valuable, but the limits of the comparison should be acknowledged. Using a newly generated pllp allele as a reference is a strong part of the paper. It gives the whole story much more weight, because it provides a plausible representation of the original mutant phenotype before long-term adaptation or drift.

At the same time, the manuscript could be more explicit about the limits of this comparison. The two lines are being used to support fairly strong conclusions, but it is hard to know how much of the difference reflects adaptation in the older line versus other background-related variables. I am not saying the comparison is invalid — only that the interpretation should be a bit more measured. A short discussion of this issue in the text would help.

5. Some of the broader claims should be scaled back. The paper is at its strongest when it stays close to the actual system: a zebrafish intestinal mutant line that appears to have changed over time in a reproducible and biologically meaningful way. Where it becomes less convincing is when the discussion broadens into more general claims about adaptive mechanisms or evolutionary implications.

I would recommend keeping the conclusions closer to the data. The study already has a solid and interesting story without needing to overextend its scope. In my view, a more restrained discussion would actually improve the paper and make the main findings easier to appreciate.

Minor points

1. Some conclusions read more strongly than the data justify. In a few places, especially in the Results, the wording is a bit too definitive. This is most noticeable when the manuscript moves from describing a phenotypic difference to assigning a mechanism. I think the paper would benefit from slightly more restrained language in those sections. The story is still interesting without pushing every observation to its strongest possible interpretation.

2. The distinction between “adapted” and “non-adapted” lines should be handled more carefully in the text. The terminology is understandable, but after a while, it starts to sound as though the nature of the difference has already been settled. Since the basis of that difference is still part of the interpretation, it may be better to remind the reader from time to time that these are operational terms rather than fully established categories. A small adjustment in wording would help avoid overstatement.

3. The transcriptomic section is harder to follow than it needs to be. The RNA-seq analysis contains useful information, but the way it is presented feels somewhat dense. It would help to guide the reader more clearly through the logic of the analysis: what was compared, what came out of those comparisons, and which changes the authors consider most relevant to the phenotype. Right now, some of the interpretation arrives a bit too quickly.

4. Immune-related changes are interesting, but the discussion could be more balanced. The immune signature is one of the more intriguing aspects of the paper, but at present, it is difficult to know how central it is to the phenotype. The manuscript sometimes gives the impression that these changes may be mechanistically important, but the evidence here is still fairly indirect. I would suggest discussing this as a plausible component of the adapted state rather than as a defined driver.

5. A few figure legends could be more explicit. In several places, I had to go back and check exactly what was being quantified and from which samples. This is not a major issue, but the figure legends could do a better job of stating the experimental comparison more directly. It would also help if the legends made clear when results come from biological replicates, pooled samples, or representative images.

Reviewer #3: This manuscript examines how zebrafish carrying loss-of-function mutations in pllp recover survival and intestinal protein uptake over multiple generations despite persistence of the original deleterious lesion. Using comparisons between an adapted historical allele (pd1116) and a more recently generated non-adapted allele (pd1245), the authors combine survival studies, RNA-seq, co-expression network analysis, and functional uptake assays to argue that adaptation is associated with increased expression of lysosome-rich enterocyte (LRE) endocytic machinery and coordinated immune remodeling. Overall, this is a compelling and technically strong study addressing an interesting biological question. The central observations are convincing and supported by multiple orthogonal approaches. Specific suggestions are provided below.

1. The manuscript convincingly demonstrates recovery of survival and growth in the adapted pd1116 line, increased expression of LRE endocytic machinery, enhanced protein uptake and degradation and coordinated transcriptional changes associated with this phenotype. However, the mechanistic basis of this adaptation remains unresolved. The discussion currently intermixes observed transcriptional remodeling and speculative mechanisms (NMD-driven transcriptional adaptation, epigenetic inheritance). These should be more clearly separated. (e.g. the data support a stable adapted transcriptional state, while the molecular mechanism underlying its emergence and inheritance remains unknown.) Ideas should be clearly framed as hypotheses to avoid over-interpretation relative to the data.

2. The use of WGCNA is appropriate and identifies modules associated regulatory programs without additional validation of genotype and diet. However, these modules are correlative and should not be interpreted as causal. Statements implying that modules represent causal regulatory programs could be softened (i.e. it might be more accurate to describe them as coordinated transcriptional programs associated with the adapted state.)

3. The recovery of survival across generations could plausibly reflect selection of several genetic modifiers of small effect during inbreeding, rather than (or in addition to) transcriptional reprogramming. The authors note that the crosses reported in Figure 6D do not support the presence of a single genetic modifier, but they should consider in the discussion the possibility of multiple genetic modifiers, particularly given the multigenerational timescale, the gradual emergence of the phenotype and the absence of direct evidence for epigenetic inheritance.

4. The authors could consider describing the pllp adaptation as a process of adaptive transcriptional remodeling under persistent selection. In this view, loss of pllp imposes chronic physiological stress, and over multiple generations, individuals with regulatory states that enhance protein uptake and balance immune responses are favored. This results in a stable lineage characterized by coordinated transcriptional changes in endocytic, metabolic, and immune pathways. The molecular basis of this stability (genetic, epigenetic, or both) remains unresolved and should be presented as an open question.

Minor suggestions

5. The main text describes 6/13 co-expression modules as ‘significant and biologically interpretable associations’ [203-204], yet Fig 2D shows 8/13 color-coded modules as significant in at least one category. A brief explanation of why 2/8 significant co-expression modules did not contribute biologically interpretable associations would be helpful.

6. Use ‘suggests’ rather than ‘indicates’ or ‘implies’ for hypothesis generating observations derived from co-expression network analysis (e.g. [232-234] “enrichment for PPAR signaling , retinol metabolism , and xenobiotic metabolism indicates that pd1116-/- has selectively suppressed certain detoxification and lipid-sensing programs.”)

7. It should be clarified that the finding that the pd1116 allele confers a higher protein absorption capacity is only correlated with transcriptional upregulation of endocytic machinery genes, albeit supported by LRE numbers. [308-312] “We found that pd1116-/- was able to both take up significantly more mTurquoise protein cargo within 30 min post-gavage (PG) and degrade them much faster than EK or pd1245-/- over a 60 min period (Figure 4B-E). Together, these data show that transcriptional upregulations of key LRE endocytic machinery genes rather than changes in LRE numbers confer a higher protein absorption capacity to the adapted pd1116 allele.”

8. Using full gene names, rather than abbreviations, would improve clarity for readers unfamiliar with these specific genes. Additionally, gene lists associated with specific processes should be supported with appropriate citations.

9. Mean bars of the ‘black’ module in Fig S2 should be colored white (or similar) for visibility.

**********

**Have all data underlying the figures and results presented in the manuscript been provided?**

Large-scale datasets should be made available via a public repository as described in the *PLOS Genetics*  data availability policy, and numerical data that underlies graphs or summary statistics should be provided in spreadsheet form as supporting information.

Reviewer #1: Yes

Reviewer #2: Yes

Reviewer #3: Yes

**********

PLOS authors have the option to publish the peer review history of their article (what does this mean?). If published, this will include your full peer review and any attached files.

If you choose “no”, your identity will remain anonymous but your review may still be made public.

**Do you want your identity to be public for this peer review?**  For information about this choice, including consent withdrawal, please see our Privacy Policy.

Reviewer #1: No

Reviewer #2: **Yes:**  Fernando Martin-Belmonte

Reviewer #3: No

[NOTE: If reviewer comments were submitted as an attachment file, they will be attached to this email and accessible via the submission site. Please log into your account, locate the manuscript record, and check for the action link "View Attachments". If this link does not appear, there are no attachment files.]

**Figure resubmission:**

**Reproducibility:**

To enhance the reproducibility of your results, we recommend that authors of applicable studies deposit laboratory protocols in protocols.io, where a protocol can be assigned its own identifier (DOI) such that it can be cited independently in the future. Additionally, PLOS ONE offers an option to publish peer-reviewed clinical study protocols. Read more information on sharing protocols at https://plos.org/protocols?utm_medium=editorial-email&utm_source=authorletters&utm_campaign=protocols

# Author comment: Author response to Decision Letter 1

**DOI:** 10.1371/journal.pgen.1012071.r002

7 Aug 2026

Attachment Submitted filename: responses to reviewers.docx

# Peer review: Decision Letter 1

**DOI:** 10.1371/journal.pgen.1012071.r003

**Authors:** Mary Mullins, Fengwei Yu

10 Sep 2026

PGENETICS-D-26-00186R1

Genome wide transcriptional changes underlie gradual and recurrent adaptation to protein malnutrition in zebrafish

PLOS Genetics

Dear Michel,

Thank you for submitting your manuscript to PLOS Genetics. As you will read, the reviewers and the editors are enthusiastic about your revised manuscript and we would be happy to accept it after you address the minor textual points made by Reviewer #2.  Therefore, we invite you to submit a revised version of the manuscript that addresses the points.

Please submit your revised manuscript within by Oct 10 2026 11:59PM. If you will need more time than this to complete your revisions, please reply to this message or contact the journal office at plosgenetics@plos.org. When you're ready to submit your revision, log on to https://www.editorialmanager.com/pgenetics/ and select the 'Submissions Needing Revision' folder to locate your manuscript file.

Please include the following items when submitting your revised manuscript:

* A letter that responds to each point raised by the editor and reviewer(s). You should upload this letter as a separate file labeled 'Response to Reviewers'. This file does not need to include responses to formatting updates and technical items listed in the 'Journal Requirements' section below.

* A marked-up copy of your manuscript that highlights changes made to the original version. You should upload this as a separate file labeled 'Revised Manuscript with Track Changes'.

* An unmarked version of your revised paper without tracked changes. You should upload this as a separate file labeled 'Manuscript'.

If you would like to make changes to your financial disclosure, competing interests statement, or data availability statement, please make these updates within the submission form at the time of resubmission. Guidelines for resubmitting your figure files are available below the reviewer comments at the end of this letter.

As the corresponding author, your ORCID iD is verified in the submission system and will appear in the published article. PLOS supports the use of ORCID, and we encourage all coauthors to register for an ORCID iD and use it as well. Please encourage your coauthors to verify their ORCID iD within the submission system before final acceptance, as unverified ORCID iDs will not appear in the published article. Only the individual author can complete the verification step; PLOS staff cannot verify ORCID iDs on behalf of authors.

We look forward to receiving your revised manuscript.

Kind regards,

Mary

Mary C. Mullins

Academic Editor

PLOS Genetics

Fengwei Yu

Section Editor

PLOS Genetics

Aimée Dudley

Editor-in-Chief

PLOS Genetics

Anne Goriely

Editor-in-Chief

PLOS Genetics

**Journal Requirements:**

If the reviewer comments include a recommendation to cite specific previously published works, please review and evaluate these publications to determine whether they are relevant and should be cited. There is no requirement to cite these works unless the editor has indicated otherwise.

1) Please amend your detailed Financial Disclosure statement. This is published with the article. It must therefore be completed in full sentences and contain the exact wording you wish to be published.

i) State what role the funders took in the study. If the funders had no role in your study, please state: "The funders had no role in study design, data collection and analysis, decision to publish, or preparation of the manuscript."

ii) If any authors received a salary from any of your funders, please state which authors and which funders..

If you did not receive any funding for this study, please simply state: u201cThe authors received no specific funding for this work.u201d

**Reviewers' comments:**

Reviewer's Responses to Questions

**Comments to the Authors:**

**Please note here if the review is uploaded as an attachment.**

Reviewer #1: The revised manuscript thoroughly addressed both major and minor concerns. They performed the RNAseq analysis that was requested on the adapted pd1245 mutants --- this nicely adds to their story. They also nicely addressed the concerns of the other reviewers specifically by softening the language about mechanisms driving the adaptive phenotypes.

Reviewer #2: I think the manuscript has improved considerably after revision. The authors have dealt with the main points raised in my previous review, and the current version is much clearer in how it presents the adaptive phenotype and the transcriptomic data. I also think the discussion is now more appropriately cautious about what can and cannot be concluded from the comparison between the two alleles. The changes made to the interpretation of the LRE and immune phenotypes are also helpful.

I only have a few minor comments before publication.

1. A few statements in the Abstract still sound somewhat more causal than the revised Discussion. For example, “resulting in a capacity for protein absorption that exceeds that of WT”, “is aided by a fine-tuned transcriptional regulation of immune genes”, and the final statement that genome-wide transcriptional changes “underlie” the stable improvements could be softened slightly. I would suggest wording such as “associated with” or “accompanied by”, which would be more consistent with the careful interpretation adopted elsewhere in the revised manuscript.

2. Similarly, at the beginning of the Discussion, the authors refer to “two intertwined adaptive mechanisms”. This still feels a little stronger than the evidence, particularly for the immune component, which is later—and appropriately—described as having an indirect relationship with improved survival. I would simply align the wording in this opening paragraph with the more cautious language used later in the Discussion.

3. There is a small typo in the Results: “fine-fined tuned regulation” should be corrected.

These are minor textual points only. In my view, the authors have responded well to the substantive issues raised in the first round, and I have no further major concerns.

Reviewer #3: The authors have addressed the reviewers' comments in a satisfactory manner.

**********

**Have all data underlying the figures and results presented in the manuscript been provided?**

Large-scale datasets should be made available via a public repository as described in the *PLOS Genetics*  data availability policy, and numerical data that underlies graphs or summary statistics should be provided in spreadsheet form as supporting information.

Reviewer #1: Yes

Reviewer #2: Yes

Reviewer #3: Yes

**********

PLOS authors have the option to publish the peer review history of their article (what does this mean?). If published, this will include your full peer review and any attached files.

If you choose “no”, your identity will remain anonymous but your review may still be made public.

**Do you want your identity to be public for this peer review?**  For information about this choice, including consent withdrawal, please see our Privacy Policy.

Reviewer #1: No

Reviewer #2: **Yes:**  Fernando Martin-Belmonte

Reviewer #3: No

[NOTE: If reviewer comments were submitted as an attachment file, they will be attached to this email and accessible via the submission site. Please log into your account, locate the manuscript record, and check for the action link "View Attachments". If this link does not appear, there are no attachment files.]

**Figure resubmission:**

**Reproducibility:**

To enhance the reproducibility of your results, we recommend that authors deposit laboratory protocols in protocols.io, where a protocol can be assigned its own identifier (DOI) such that it can be cited independently in the future. Additionally, PLOS ONE offers an option to publish peer-reviewed clinical study protocols. Read more information on sharing protocols at https://plos.org/protocols?utm_medium=editorial-email&utm_source=authorletters&utm_campaign=protocols

# Author comment: Author response to Decision Letter 2

**DOI:** 10.1371/journal.pgen.1012071.r004

11 Sep 2026

Attachment Submitted filename: responses to reviewers 2.docx

# Editor report: Decision Letter 2

**DOI:** 10.1371/journal.pgen.1012071.r005

**Authors:** Mary Mullins, Fengwei Yu

17 Sep 2026

Dear Michel,

We are pleased to inform you that your manuscript entitled "Genome wide transcriptional changes underlie gradual and recurrent adaptation to protein malnutrition in zebrafish" has been editorially accepted for publication in PLOS Genetics. Congratulations!

Before your submission can be formally accepted and sent to production you will need to complete our formatting changes, which you will receive in a follow up email. Please be aware that it may take several days for you to receive this email; during this time no action is required by you. Please note: the accept date on your published article will reflect the date of this provisional acceptance, but your manuscript will not be scheduled for publication until the required changes have been made.

Once your paper is formally accepted, an uncorrected proof of your manuscript will be published online ahead of the final version, unless you’ve already opted out via the online submission form. If, for any reason, you do not want an earlier version of your manuscript published online or are unsure if you have already indicated as such, please let the journal staff know immediately at plosgenetics@plos.org.

In the meantime, please log into Editorial Manager at https://www.editorialmanager.com/pgenetics/, click the "Update My Information" link at the top of the page, and update your user information to ensure an efficient production and billing process. Note that PLOS requires an ORCID iD for all corresponding authors. Therefore, please ensure that you have an ORCID iD and that it is validated in Editorial Manager. To do this, go to ‘Update my Information’ (in the upper left-hand corner of the main menu), and click on the Fetch/Validate link next to the ORCID field.  This will take you to the ORCID site and allow you to create a new iD or authenticate a pre-existing iD in Editorial Manager.

If you have a press-related query, or would like to know about making your underlying data available (as you will be aware, this is required for publication), please see the end of this email. If your institution or institutions have a press office, please notify them about your upcoming article at this point, to enable them to help maximise its impact. Inform journal staff as soon as possible if you are preparing a press release for your article and need a publication date.

Thank you again for supporting open-access publishing; we are looking forward to publishing your work in PLOS Genetics!

Best,

Mary

Mary C. Mullins

Academic Editor

PLOS Genetics

Fengwei Yu

Section Editor

PLOS Genetics

Aimée Dudley

Editor-in-Chief

PLOS Genetics

Anne Goriely

Editor-in-Chief

PLOS Genetics

www.plosgenetics.org

BlueSky: @plos.bsky.social

----------------------------------------------------

Comments from the reviewers (if applicable):

----------------------------------------------------

**Data Deposition**

If you have submitted a Research Article or Front Matter that has associated data that are not suitable for deposition in a subject-specific public repository (such as GenBank or ArrayExpress), one way to make that data available is to deposit it in the Dryad Digital Repository. As you may recall, we ask all authors to agree to make data available; this is one way to achieve that. A full list of recommended repositories can be found on our website.

The following link will take you to the Dryad record for your article, so you won't have to re‐enter its bibliographic information, and can upload your files directly:

http://datadryad.org/submit?journalID=pgenetics&manu=PGENETICS-D-26-00186R2

More information about depositing data in Dryad is available at http://www.datadryad.org/depositing. If you experience any difficulties in submitting your data, please contact help@datadryad.org for support.

Additionally, please be aware that our data availability policy requires that all numerical data underlying display items are included with the submission, and you will need to provide this before we can formally accept your manuscript, if not already present.

----------------------------------------------------

**Press Queries**

If you or your institution will be preparing press materials for this manuscript, or if you need to know your paper's publication date for media purposes, please inform the journal staff as soon as possible so that your submission can be scheduled accordingly. Your manuscript will remain under a strict press embargo until the publication date and time. This means an early version of your manuscript will not be published ahead of your final version. PLOS Genetics may also choose to issue a press release for your article. If there's anything the journal should know or you'd like more information, please get in touch via plosgenetics@plos.org.

# Editor report: Acceptance letter

**DOI:** 10.1371/journal.pgen.1012071.r006

**Authors:** Mary Mullins, Fengwei Yu

PGENETICS-D-26-00186R2

Genome wide transcriptional changes underlie gradual and recurrent adaptation to protein malnutrition in zebrafish

Dear Dr Bagnat,

We are pleased to inform you that your manuscript entitled "Genome wide transcriptional changes underlie gradual and recurrent adaptation to protein malnutrition in zebrafish" has been formally accepted for publication in PLOS Genetics! Your manuscript is now with our production department and you will be notified of the publication date in due course.

The corresponding author will soon be receiving a typeset proof for review, to ensure errors have not been introduced during production. Please review the PDF proof of your manuscript carefully, as this is the last chance to correct any errors. Please note that major changes, or those which affect the scientific understanding of the work, will likely cause delays to the publication date of your manuscript.

Soon after your final files are uploaded, unless you have opted out or your manuscript is a front-matter piece, the early version of your manuscript will be published online. The date of the early version will be your article's publication date. The final article will be published to the same URL, and all versions of the paper will be accessible to readers.

For Research Articles, you will receive an invoice from PLOS for your publication fee after your manuscript has reached the completed accept phase. If you receive an email requesting payment before acceptance or for any other service, this may be a phishing scheme. Learn how to identify phishing emails and protect your accounts at https://explore.plos.org/phishing.

Thank you again for supporting PLOS Genetics and open-access publishing. We are looking forward to publishing your work!

With kind regards,

Janani Seenivasan

PLOS Genetics

On behalf of:

The PLOS Genetics Team

Carlyle House, Carlyle Road, Cambridge CB4 3DN | United Kingdom

plosgenetics@plos.org | +44 (0) 1223-442823

plosgenetics.org | Twitter: @PLOSGenetics
